# Astrocyte-Derived PTPRZ1 Regulates Excitatory Synapse Density in the Mouse Cortex

**DOI:** 10.1523/ENEURO.0386-25.2026

**Published:** 2026-04-21

**Authors:** Alex R. Eaker, Hayli E. Spence-Osorio, Madelyn G. Coble, Breana C. Dogan, Katherine T. Baldwin

**Affiliations:** ^1^ Neuroscience Center, University of North Carolina at Chapel Hill, Chapel Hill, North Carolina 27599; ^2^ Departments of Psychology and Neuroscience, University of North Carolina at Chapel Hill, Chapel Hill, North Carolina 27599; ^3^ Cell Biology and Physiology, University of North Carolina at Chapel Hill, Chapel Hill, North Carolina 27599

**Keywords:** astrocyte, development, PTPRZ1, synapse

## Abstract

Protein tyrosine phosphatase receptor type Z1 (*Ptprz1*) is one of the most abundantly expressed and enriched genes in astrocytes during development, yet its function in astrocytes is unknown. Using an astrocyte–neuron coculture system, we found that knockdown of *Ptprz1* in astrocytes significantly impaired astrocyte branching morphogenesis. To investigate the function of *Ptprz1* in astrocytes during brain development, we generated a *Ptprz1* conditional knock-out mouse and deleted *Ptprz1* from astrocytes postnatally, after the bulk of astrogenesis is complete. At postnatal day 21, we found subtle changes in astrocyte morphology and a reduction in the density of colocalized pre- and postsynaptic excitatory synapse markers across multiple layers of the visual cortex in both male and female mice, suggesting important functions for astrocytic *Ptprz1* in both astrocyte morphogenesis and synaptogenesis. *Ptprz1* is expressed in several neural cell types, including radial glial stem cells and oligodendrocyte progenitor cells, and regulates critical aspects of neurodevelopment, including neurite outgrowth, neuronal differentiation, myelination, and extracellular matrix development. Moreover, altered *Ptprz1* expression is associated with schizophrenia and glioblastoma. Therefore, this mouse model is a valuable resource for investigating cell-type-specific *Ptprz1* function in numerous neurodevelopmental and neuropathological mechanisms.

## Significance Statement

Protein tyrosine phosphatase receptor type Z1 (PTPRZ1) is an abundant, astrocyte-enriched protein linked to neurological dysfunction; however, its astrocyte-specific functions are unknown. We generated a *Ptprz1* conditional knock-out mouse and found that astrocyte-specific deletion of *Ptprz1* reduces the density of colocalized excitatory synapse markers in the developing mouse cortex, with mild impact to astrocyte morphology. PTPRZ1 is an emerging therapeutic target for glioblastoma and neurodegeneration. This study provides a new tool to study PTPRZ1 function in neurodevelopment and neuropathology.

## Introduction

Astrocytes are morphologically complex glial cells that control many critical aspects of central nervous system function, including synapse formation, neurovascular coupling, and ion and fluid homeostasis. In the mouse cortex, the bulk of astrocyte morphogenesis occurs during the second and third postnatal weeks, coinciding with a period of peak synaptogenesis (Stogsdill et al., 2017; Clavreul et al., 2019; Lee et al., 2025; Rodriguez Salazar et al., 2025). Astrocytes express numerous membrane-bound proteins in a developmentally regulated manner to interact with both secreted factors and cell adhesion molecules. These interactions facilitate bidirectional communication between astrocytes and neurons and promote structural and functional development in both astrocytes and neuronal synapses (Stogsdill et al., 2017; Xie et al., 2022; Cheng et al., 2023). While the list of molecules involved in astrocyte development continues to expand, substantial knowledge gaps still exist, and the function of many proteins that are highly expressed in astrocytes during development is unknown.

Protein tyrosine phosphatase receptor Z1 (*Ptprz1*) is one of the most abundantly expressed genes in cortical astrocytes during development and is strongly enriched in astrocytes compared with all other brain cell types (Zhang et al., 2014; Zhang et al., 2016); however, its function in astrocytes is unknown. *Ptprz1* encodes for protein tyrosine phosphatase receptor type Z1 (PTPRZ1), a member of the receptor-like protein tyrosine phosphatase family. Three splice isoforms of PTPRZ1 have been characterized, including membrane-bound long and short isoforms and a secreted isoform known as phosphacan (Maurel et al., 1994). Both the long isoform and phosphacan are chondroitin sulfate proteoglycans (CSPG), whereas the short isoform has been detected in both CSPG and non-CSPG form (Maurel et al., 1994; Nishiwaki et al., 1998). Immunoblotting of rodent brain lysates indicates that all three isoforms are expressed during development (Sakurai et al., 1996; Snyder et al., 1996; Nishiwaki et al., 1998), and transcriptomic data and histological experiments confirm developmental expression in radial glial stem cells (RGCs; Loo et al., 2019), neurons (Hayashi et al., 2005b), and oligodendrocyte precursor cells (OPCs; Zhang et al., 2014; Zhang et al., 2016), albeit at lower levels than astrocytes (Zhang et al., 2014; Zhang et al., 2016; Loo et al., 2019).

Prior studies have revealed important functional roles of PTPRZ1 in multiple aspects of neurodevelopment. PTPRZ1 regulates cortical neuron migration in primary culture (Maeda and Noda, 1998) and Purkinje cell dendrite morphology in organotypic slice cultures (Tanaka et al., 2003). A number of studies have found key roles of PTPRZ1 in OPC proliferation and differentiation (McClain et al., 2012; Kuboyama et al., 2016; Tanga et al., 2019), as well as recovery from demyelinating lesions (Harroch et al., 2002; Kuboyama et al., 2015). More recently, studies with constitutive *Ptprz1* knock-out mice found that loss of *Ptprz1* impairs angiogenesis (Choleva et al., 2024) and perineuronal net structure (Eill et al., 2020; Sinha et al., 2023). Importantly, changes in PTPRZ1 expression are linked to schizophrenia (Buxbaum et al., 2008; Takahashi et al., 2011; Cressant et al., 2017), and PTPRZ1 is emerging as a therapeutic target for the treatment of glioblastoma (Fujikawa et al., 2016; Fujikawa et al., 2017; Papadimitriou and Kanellopoulou, 2023; Yang et al., 2023), substance use disorder (Fernandez-Calle et al., 2018; Pastor et al., 2018), and neurodegenerative conditions such as multiple sclerosis (Song et al., 2025) and Alzheimer’s disease (Fontan-Baselga et al., 2024). Though studies have suggested the role of astrocyte-expressed PTPRZ1 in neuronal function (Tanaka et al., 2003; Parent et al., 2007) and demyelination (Takahashi et al., 2023), the role of astrocytic PTPRZ1 in the aforementioned neurodevelopmental processes and neurological disorders has not been investigated. Moreover, it is unclear whether and how PTPRZ1 is important for proper development and function of astrocytes themselves.

Parsing out the cell-type-specific contributions of PTPRZ1 is challenging, due to its developmental expression in multiple cell types. To overcome this technical limitation and investigate the role of astrocyte-derived PTPRZ1 in postnatal brain development, we generated a new transgenic mouse line with a floxed *Ptprz1* allele and deleted *Ptprz1* from astrocytes postnatally. Our findings suggest roles of astrocytic PTPRZ1 in astrocyte morphogenesis and synaptogenesis and demonstrate the utility of this mouse model for investigating cell-type-specific functions of PTPRZ1.

## Materials and Methods

### Animals

All animal procedures were performed in accordance with the University of North Carolina at Chapel Hill Institutional Animal Care and Use Committee's regulations. Mice were housed in standard conditions with 12 h day/night cycles. Aldh1L1-Cre/ERT2 BAC transgenic (RRID:IMSR_JAX:029655), ROSA-td-Tomato Ai14 (RTM; RRID:IMSR_JAX:007914), FLPo (RRID:IMSR_JAX:012930), and C57BL/6J (RRID:IMSR_JAX:000664) lines were obtained through Jackson Laboratory. Aldh1l1-GFP transgenic mice were obtained from MMRRC (RRID:MMRRC_011015-UCD).

*Ptprz1* conditional knock-out (cKO) mice were generated using homologous recombination. Briefly, a bacterial artificial chromosome (BAC) with homology to the *Ptprz1* genomic locus was generated to insert *loxP* sites before Exon 5 and after Exon 6. BACs were injected into mouse ES cells (G4-129S6B6F1; RRID:CVCL_E222), and positive clones were injected into pseudopregnant females. Chimeric offspring males were mated to C57BL/6J females to achieve germline transmission, identified via PCR amplification of genomic DNA using the following primers: forward 5′ TCACAAGGGTTAGCTTCACAG 3′ and reverse 5′ AGCAGTAGACTTGCATCTGTG 3′ [wild type (WT), 644 bp; Flox, 733 bp]. Mice with germline transmission were mated with *Flpo* transgenic mice on a C57BL/6J background to excise the neomycin selection cassette. Recombined offspring were mated with C57BL/6J, RTM, or Aldh1L1-CreERT2 transgenic mice to generate breeding pairs for experiments.

Mice were assigned to experimental groups based on genotype and collected for experiments at Postnatal Day 7 (P7), P14, and P21. All mice used in experiments expressed exactly one copy of the Aldh1L1-CreERT2 transgene. For synapse analysis, same-sex littermate pairs were collected at P21. For all experiments, mice of both sexes were included in analysis. Criteria for inclusion, exclusion, and randomization are listed for each experiment in specific method subsections.

### Cell culture

#### Cortical neurons

Purified rat cortical neurons were isolated by immunopanning for neuronal L1CAM. Briefly, cortices were microdissected from P1 rat pups of both sexes, digested in papain (7.5 units/ml; Worthington Biochemical, LK003178), triturated in low and high ovomucoid (Worthington Biochemical LS003086) solutions, resuspended in panning buffer [DPBS (Invitrogen 14287) supplemented with BSA (Sigma-Aldrich A2153-50G) and insulin (Sigma-Aldrich I1882-100MG)], and passed through a 20 µm mesh filter (Elko Filtering 03-20/14). Filtered cells were incubated on negative panning dishes coated with Bandeiraea Simplicifolia Lectin 1, followed by goat anti-mouse IgG + IgM (H + L; Jackson ImmunoResearch Laboratories115-005-044; RRID:AB_2338451) and goat anti-rat IgG + IgM (H + L; Jackson ImmunoResearch Laboratories 112-005-044; RRID:AB_2338094) antibodies, and then incubated on positive panning dishes coated with mouse anti-L1 (ASCS4, Developmental Studies Hybridoma Bank, University of Iowa). Adherent cells were dislodged using a P1000 pipet, pelleted (11 min at 200 rcf), and resuspended in serum-free neuron growth media [NGM; Neurobasal (Thermo Fisher Scientific 21103049), B-27 supplement (Thermo Fisher Scientific 17504044), 2 mM l-glutamine (Life Technologies 25030081), 100 U/ml pen/strep (Life Technologies 15140122), 1 mM sodium pyruvate (Life Technologies 11360070), 4.2 µg/ml Forskolin (Sigma-Aldrich F6886-25MG), 50 ng/ml BDNF (PeproTech 450-02), and 10 ng/ml CNTF (PeproTech 450-13)]. The 70,000 neurons were plated onto 12 mm glass coverslips coated with 10 µg/ml poly-d-lysine (PDL; Sigma-Aldrich P6407) and 2 µg/ml laminin and incubated at 37°C in 10% CO_2_. On day in vitro (DIV) 2, half of the media was replaced with NGM and 10 µM AraC (C1768-100MG). On DIV 3, the media was replaced with NGM. Half of the media was replaced with NGM on DIV 6 and DIV 9.

#### Cortical astrocytes

P1 rat cortices from both sexes were microdissected, digested in papain, triturated in low and high ovomucoid solutions, filtered, and resuspended in astrocyte growth media [AGM; DMEM (Invitrogen 11960), 10% FBS (Life Technologies A5670801), 10 µM hydrocortisone (Sigma-Aldrich H0888-5G), 100 U/ml pen/strep, 2 mM l-glutamine, 5 µg/ml insulin, 1 mM Na pyruvate (Life Technologies 11360070), 5 µg/ml *N*-acetyl-l-cysteine (Sigma-Aldrich A8199-10G)]. Between 15 and 20 million cells were plated on 75 mm^2^ flasks (nonventilated cap) coated with PDL (Sigma-Aldrich P10224-10MG) and incubated at 37°C in 10% CO_2_. On DIV 3, nonastrocyte cells were removed by forceful shaking of closed flasks. AraC was added on DIV 5 to eliminate fibroblasts. On DIV 7, astrocytes were trypsinized (0.05% trypsin-EDTA; Life Technologies 25300054) and plated into 12-well (200,000 cells/well) or 6-well (400,000 cells/well) plates. On DIV 8, cultured rat astrocytes were transfected with shRNA plasmids using Lipofectamine LTX with Plus Reagent (Thermo Fisher Scientific 15338030). Briefly, 1 µg (12-well) or 2 µg (6-well) total DNA was diluted in Opti-MEM (Thermo Fisher Scientific 11058021) containing Plus Reagent, mixed with Opti-MEM containing LTX (1:2 DNA to LTX) and incubated for 30 min at room temperature. The transfection solution was added to astrocyte cultures and incubated at 37C for 3 h and then replaced with AGM. On DIV 10, astrocytes were trypsinized, resuspended in NGM, plated (20,000 cells per well) onto DIV 10 neurons in serum-free media, and cocultured for 48 h.

#### HEK293T

HEK293T cells (University of North Carolina at Chapel Hill Tissue Culture Facility; RRID:CVCL_0063) used to produce lentivirus and adeno-associated virus (AAV) were cultured in DMEM (GIBCO 11960) supplemented with 10% FBS, 100 U/ml pen/strep, 2 mM l-glutamine, and 1 mM sodium pyruvate. Cells were incubated at 37°C in 5% CO_2_ and passaged every 2–3 d.

### Plasmids

pLKO.1 Puro plasmids containing shRNA against mouse/rat *Ptprz1* were obtained from the RNAi Consortium (TRC) via Dharmacon (Clone ID, TRCN0000081069; shRNA sequence, CTCCTTAAACAGTGGCTCTAA). A scrambled shRNA sequence was generated by annealing the following oligonucleotides:

Fwd: CCGGGATAACCGTATTCACGCTATCCTCGAGGATAGCGTGAATACGGTTATCTTTTT

G Rev: AATTCAAAAAGATAACCGTATTCACGCTATCCTCGAGGATAGCGTGAATACGGTTATC

and cloned into the pLKO.1 Puro TRC cloning vector at AgeI and EcoRI restriction sites according to Addgene protocols. pLKO.1 shRNA plasmids expressing CAG-GFP in place of the puromycin resistance gene were generated by restriction enzyme cloning at KpnI and SpeI sites.

### Lentivirus production and transduction

Lentiviruses containing shRNA targeting vectors were produced by transfecting HEK293T cells with pLKO.1 shRNA-Puro, VSVG, and dR8.91 using X-tremeGENE (Sigma-Aldrich 8724105001). The following day, media were replaced with AGM, and media containing lentivirus was collected on days 2 and 3 post-transfection. To test the knockdown efficiency of *Ptprz1* shRNA, DIV 7 rat primary cortical astrocytes were trypsinized and plated into 6-well plates (400,000 cells/well) in 2 ml of AGM. On DIV 8, 1 ml of AGM was removed from the astrocytes and replaced with 1 ml of media containing the following: 500 µl fresh AGM, 500 µl lentivirus-containing media, and 1 µg/ml polybrene (Sigma-Aldrich H9268-5G). Cultures were treated with puromycin (1 µg/ml) from DIV 10–15 to eliminate nontransduced cells. On DIV 15, protein was extracted using membrane solubilization buffer (25 mM Tris, 150 mM NaCl, 1 mM CaCl2, 1 mM MgCl_2_, 0.5% NP-40, and protease inhibitors), pH 7.4.

### Immunocytochemistry

DIV 12 cocultures were incubated with warm 4% paraformaldeyhde (PFA) for 7 min, washed three times with phosphate-buffered saline (PBS), and blocked in PBS containing 50% normal goat serum (NGS; Thermo Fisher Scientific) and 0.4% Triton X-100 (Sigma-Aldrich) for 30 min at room temperature. Samples were washed once more in PBS and incubated overnight at 4°C with chicken anti-GFP (Aves GFP1020, 1:1,000; RRID:AB_10000240) diluted in antibody blocking buffer (ABB; 150 mM NaCl, 50 mM Tris, 1% BSA, 100 mM l-lysine, 0.04% sodium azide), pH 7.4, containing 10% NGS. The following day, samples were washed three times with PBS, incubated with goat anti-chicken IgY Alexa Fluor 488 (Life Technologies, 1:500; RRID: AB_2534096) diluted in ABB with 10% NGS for 2 h at room temperature, and washed again three times in PBS. Coverslips were mounted onto glass slides using Vectashield mounting media with DAPI (Vector Labs) and sealed with nail polish. Healthy astrocytes with strong expression of GFP, a single nucleus, and minimal overlap with other GFP^+^ astrocytes were imaged at 40× magnification in green and DAPI channels using a Zeiss AxioImager M1. The individual acquiring the images was always blinded to the experimental condition. Sholl analysis was performed in Fiji (Plugin, Sholl_Analysis-3.7.4.jar) and statistical analysis performed in RStudio using a linear mixed model with Tukey’s HSD. At least 20 cells were imaged per condition per experiment, from three independent experiments. Astrocytes containing multiple nuclei, weak GFP expression, or in areas of dense GFP-labeled cells were not imaged. For experiments in which the peak of the shScr control condition Sholl analysis curve failed to reach 18 intersections, all conditions were excluded from analysis due to suboptimal neuron health. If the peak of the shScr control condition Sholl analysis curve reached 18 or above, then all conditions were included in analysis.

### AAV production and administration

To produce purified AAV, pZac2.1-gfaABC1D-GFP-CAAX HEK293T cells were transfected with pAD-DELTA F6, serotype plasmid AAV PHP.eB, and pZac2.1-gfaABC1D-GFP-CAAX. Three days after transfection, cells were collected and lysed and AAV-enriched fraction isolated from the supernatant by Optiprep density gradient and ultracentrifugation. AAV containing pZac2.1-GfaABC1D-mCherry CAAX was produced by the BRAIN Initiative Viral Vector Core. Briefly, purified AAVs were exchanged into storage buffer containing 1× PBS, 5% d-sorbitol, and 350 mM NaCl. Virus titers (GC/milliliter) were determined by qPCR targeting the AAV inverted terminal repeats. To label astrocytes with GFP-CAAX or mCherry-CAAX, 1 µl of AAV was injected unilaterally into the cortex of hypothermia-anesthetized P2 neonates using a Hamilton syringe.

### Tamoxifen administration

Tamoxifen was administered via intragastric injection at P2 and P3. Tamoxifen powder (Sigma-Aldrich T5648-1G) was dissolved in corn oil at 10 mg/ml and further diluted in corn oil to 1.25 mg/ml (for P2 injection) and 2.5 mg/ml (for P3 injection). A 40 µl of the respective tamoxifen solution was injected into the milk spot using an insulin syringe, for a dose of 0.05 mg at P2 and 0.1 mg at P3.

### Immunohistochemistry

#### Sample preparation

Mice were anesthetized with 0.8 mg/kg tribromoethanol (Avertin) and perfused with tris-buffered saline (TBS)/heparin, followed by 4% PFA in TBS. Brains were postfixed overnight in 4% PFA, rinsed three times with TBS, and cryoprotected in 30% sucrose in TBS. Brains were frozen in embedding molds using a medium containing two parts 30% sucrose and one part O.C.T. and then stored at −80°C. Frozen brains were sectioned coronally to 25, 40, or 100 µm thickness on a CryoStar NX50 Cryostat (Thermo Fisher Scientific) and stored in a 1:1 mixture of glycerol and 1× TBS at −25°C until use. For immunostaining, sections were washed in TBST (0.2% Triton X-100 in TBS), blocked in blocking solution (10% NGS in TBS + 0.2% Triton X-100 [TBST]), and incubated in primary antibody solution (primary antibody diluted in blocking solution) for 2–3 nights at 4°C while shaking at 100 rpm (see specific subsections below for antibody concentrations). Following primary antibody incubation, sections were washed in TBST, incubated in secondary antibody solution (secondary antibody diluted 1:200 in blocking solution) for 2–3 h at room temperature, and washed again in TBST. DAPI was added to the secondary antibody solution for the final 10 min of incubation at a 1:50,000 concentration. Sections were then mounted onto glass slides with homemade mounting medium (20 mM Tris pH 8.0, 90% glycerol, 0.5% *N*-propyl gallate), and sealed with nail polish. For primary antibodies produced in mouse, isotype subgroup-specific secondary antibodies were used (e.g., goat anti-mouse IgG1) to prevent excessive background staining; the isotype subgroup is specified for all subgroup-specific antibodies used.

#### Validation of astrocyte-specific Ptprz1 deletion

PTPRZ1 expression by astrocytes was visualized using 40-µm-thick sections of P21 *Ptprz1* WT, cHet, and cKO brain tissue containing primary visual cortex (V1). Sections were labeled with guinea pig anti-RFP (Synaptic Systems 390004, 1:1,000; RRID:AB_2737052) and mouse IgG1 anti-phosphacan (clone 3F8, Developmental Studies Hybridoma Bank, University of Iowa; 1:100) followed by goat anti-guinea pig IgG Alexa Fluor 594 (Thermo Fisher Scientific A-11076, 1:200; RRID:AB_2534120) and goat anti-mouse IgG1 Alexa Fluor 488 (Thermo Fisher Scientific A-21121, 1:200; RRID:AB_2535764). Multi-tile confocal images of the entire tissue sections containing V1 were collected using the Leica Stellaris 8 FALCON STED with a 20× (0.75 NA) oil-immersion objective. High-magnification, single-tile *z*-stacks of 10–15 μm thickness in V1 layer 5 (L5) were acquired with a 100× (1.4 NA) oil-immersion objective. High-magnification data represent maximum intensity projections of three optical sections per channel per group to assess phosphacan localized within astrocyte cell bodies and processes. Image processing and binarized masks of phosphacan signal within astrocyte cell bodies and processes were created with Fiji/ImageJ software. PTPRZ1 expression by astrocytes was similarly visualized using 40-µm-thick sections of the P7 *Ptprz1* WT and cKO brain tissue containing V1.

PTPRZ1 expression by other cell types (OPCs and neurons) was visualized using 40-µm-thick sections of the P21-22 *Ptprz1* WT and cKO brain tissue. Sections were labeled with a combination of (1) mouse IgG1 anti-phosphacan (1:100)/goat anti-mouse IgG1 Alexa Fluor 488 (1:200) and rabbit IgG anti-PDGFRα (Cell Signaling Technology 3174S, 1:500; RRID:AB_2162345)/goat anti-rabbit IgG Alexa Fluor 647 (Thermo Fisher Scientific A-21245, 1:200; RRID:AB_2535813) to identify PTPRZ1 expression in OPCs or (2) mouse IgG1 anti-phosphacan (1:100)/goat anti-mouse IgG1 Alexa Fluor 488 (1:200) and rabbit IgG anti-β3-tubulin (Cell Signaling Technology 5568S, 1:500; RRID:AB_10694505)/goat anti-rabbit IgG Alexa Fluor 647 (1:200) to identify PTPRZ1 expression in neurons. High-magnification images were acquired as described above in V1 L5 and corpus callosum (CC). Data represent maximum intensity projections of three optical sections per channel per group; binarized masks for OPCs and CC images were prepared as described above, while region of interest (ROI)-based binarized masks were generated for neuronal cell bodies and proximal processes to specifically observe PTPRZ1 expression in neurons.

#### Cell counting

The 40-µm-thick sections containing V1 were labeled with one of two antibody combinations for (1) astrocyte and (2) neuron counting: (1) rabbit anti-Sox9 (Millipore AB5535, 1:1,000; RRID:AB_2239761)/goat anti-rabbit IgG Alexa Fluor 647, mouse IgG2a anti-Olig2 (Millipore MABN50, 1:400; RRID:AB_10807410)/goat anti-mouse IgG2a Alexa Fluor 488 (Thermo Fisher Scientific A-21131; RRID: AB_2535771), and DAPI or (2) mouse IgG1 anti-NeuN (Millipore MAB377, 1:1,000; RRID: AB_2298772)/goat anti-mouse IgG1 Alexa Fluor 488 (Thermo Fisher Scientific A-21121; RRID:AB_2535764) and DAPI. Corresponding secondary antibodies produced in goat were used at 1:200. For each staining condition, three sections per brain from six sex-matched littermate pairs (*n* = 6, three males/three females per genotype) were collected and analyzed. Tile scan images were acquired from P21 *Ptprz1* control (WT and cHet) and cKO mice using an Olympus FV3000RS inverted confocal microscope with a resonant scanner and 20× objective. For each brain section, an ROI of size 447.47 × 930.98 mm spanning L1 through L6 of the visual cortex was selected for analysis of cell number. All image processing and analysis were completed using novel, semiautomated CellProfiler pipelines (available at https://github.com/BaldwinLabUNC/Astrocyte_morphology). Images were denoised using the GaussianFilter module, then nuclei signal was enhanced and background signal was suppressed using the EnhanceOrSuppressFeatures module. DAPI^+^ nuclei were identified with the IdentifyPrimaryObjects module, and new images with the identified nuclei were saved. To identify Sox9^+^ and Olig2^+^ nuclei, the EnhanceEdges module was used to improve the identification of nuclei and help distinguish nuclei from debris, and then objects were segmented using the IdentifyPrimaryObjects module. Mean fractional intensities (MeanFrac) of the objects were measured using the MeasureObjectIntensityDistribution module, and a single maximum MeanFrac value was used for each animal in the FilterObjects module as a threshold to filter out non-nuclear objects. New images with the identified nuclei were saved, and then colocalized Sox9^+^ and Olig2^+^ nuclei were identified using the RelateObjects module and saved as new images. To identify NeuN^+^ nuclei, objects were segmented using the IdentifyPrimaryObjects module, then objects were filtered to exclude non-nuclear objects using the FilterObjects module based on eccentricity values measured in the MeasureObjectSizeShape module, and new images with the identified nuclei were saved. In all cases, the ExportToSpreadsheet module was used to count the number of objects in the saved images containing identified nuclei, and these counts were used as the Cells/Image data points. Animal averages were analyzed using a linear mixed-effects model with genotype and sex as fixed effects. To control for biological variation between litters and technical variation between imaging session, litter and imaging session were included as random effects. The model was fit using restricted maximum likelihood (REML) with the lme4 package (version 1.1-38) in R (version 2025.05.1). The reported *p* value reflects the effect of genotype, and Cohen's *d* with 95% confidence intervals reports the effect size. The experimenters were blinded to the subject group during image acquisition and analysis. All mice that appeared healthy at the time of collection were included in this study. No data were excluded.

#### Astrocyte 3D morphology analysis

Individual astrocyte territory volume and surface area was assessed in 100-μm-thick floating sections of the mouse V1 collected at P21 and P14. The tissue from *Ptprz1* WT (P21: *n* = 4, 2 males/2 females; P14: *n* = 8, 4 males/4 females), cHet (P21: *n* = 5, 1 male/4 females; P14: *n* = 8, 4 males/4 females), and cKO (P21: *n* = 7, 4 males/3 females; P14: *n* = 8, 4 males/4 females) mice with intracortical AAV injection of GFP-CAAX (P21) or mCherry-CAAX (P14) was collected, processed, and stained as described above using the following antibody combinations at (1) P21 and (2) P14: (1) chicken anti-GFP (Aves Labs GFP1010, 1:1,000; RRID:AB_2307313)/goat anti-chicken IgY Alexa Fluor 488 (Thermo Fisher Scientific A-11039, 1:200; RRID:AB_2534096) and DAPI or (2) guinea pig anti-RFP (Synaptic Systems 390004, 1:1,000; RRID:AB_2737052)/goat anti-guinea pig IgG Alexa Fluor 594 (Thermo Fisher Scientific A-11076, 1:200; RRID:AB_2534120) and DAPI. High-magnification images containing whole astrocytes (50–60 μm *z*-stack) were acquired on an Olympus FV3000 microscope with a 40× objective and 2× optical zoom. Inclusion criteria for analysis required the entirety of the astrocyte to be contained within a single brain section, specifically V1 L5. Astrocytes outside of this brain region and/or incomplete astrocytes were excluded from this study. The Imaris (Bitplane) software was used as described previously to analyze astrocyte territory volume (Eaker and Baldwin, 2022). Briefly, minimal postprocessing (median filter 3 × 3 × 3; background subtraction sigma, 40.00; normalize layers) was performed through batch processing in Imaris to aid whole-cell surface reconstruction with the surface creation tool for astrocytes labeled with eGFP-CAAX (P21). Astrocytes labeled with mCherry-CAAX were minimally postprocessed (median filter 3 × 3 × 3; background subtraction sigma, 40.00) as needed in Imaris based on quality of immunolabeling (P14). Spots close to surfaces were then generated, and a custom Convex Hull Xtension was used to build a convex hull around the whole astrocyte territory. A small number of astrocytes were excluded from the final analysis due to exceptionally dim fluorescent signal or poor subject tissue and image quality. Files containing detailed surface (astrocyte) and convex (territory) metrics were exported from Imaris for each individual astrocyte. A custom RStudio script (available at https://github.com/BaldwinLabUNC/Astrocyte_morphology) was developed to extract appropriate descriptive metrics (Area, Oblate Ellipticity, Prolate Ellipticity, Sphericity, and Volume) from exported CSV files, combine data into a single .csv file for additional statistical analysis in GraphPad Prism 10, and run preliminary statistical analyses on animal averages: the Shapiro–Wilk normality test, Levene's test for homogeneity of variance, and a subsequently appropriate one-way ANOVA with the Tukey's post-test or Kruskal–Wallis test with Dunn's multiple-comparisons test. Normality and homogeneity of variance test results were used to determine appropriate *t* tests run in GraphPad Prism 10 for plotting and *p* value reporting. Statistics using individual cell values as opposed to animal average values were conducted in GraphPad Prism 10 using the same abovementioned tests for normality and variance prior to conducting appropriate unpaired statistical tests. The experimenter was blinded to subject group during image acquisition and analysis in Imaris. The number of animals and cells/animal analyzed is indicated in the corresponding figure legend for this experiment.

#### Neuropil infiltration volume analysis

Astrocyte infiltration into the surrounding neuropil was analyzed in 40-μm-thick floating sections of P21 and P14 mouse V1 (*n* as described above, Astrocyte 3D morphology analysis). Corresponding antibody combinations used for astrocyte three-dimensional (3D) morphology analysis at P21 and P14 (see previous section) were also used for neuropil infiltration volume (NIV) analysis, with the exclusion of DAPI. High-magnification *Z*-stack images were acquired on an Olympus FV3000 microscope with a 60× objective at 2× optical zoom. Inclusion criteria required astrocytes be located in V1 L5, demonstrate sufficiently bright fluorescent labeling, encompass the entire astrocyte arbor in the X/Y plane, and include at least 10 μm of the astrocyte arbor above and below the soma in the *Z*-stack. Astrocytes failing to meet these criteria were not imaged or otherwise excluded from the final analysis. For each astrocyte, three ROIs (12.65 μm × 12.65 μm × 10 μm) containing only neuropil (excluding astrocyte soma, large branches, and end feet) were selected and reconstructed in Imaris using the surface tool. Surface volume within individual ROIs was recorded and averaged for each cell (3 × ROIs) and animal (3 × ROIs across 4–5 cells per animal). Additional astrocytes were excluded from the final analysis for being a duplicate (one cell) or twin astrocyte (one cell), decreasing the total cells from five to four for two subjects. The experimenters were blinded to subject group during image acquisition and Imaris analysis. Animal averages were analyzed a one-way ANOVA with Tukey's post-test (P21 and P14 V1 L5). Individual cell averages for NIV were analyzed using either a one-way ANOVA with Tukey's post-test (P21 V1 L5) or an unpaired two-sample *t* test (Student's *t* test; P21 V1 L1). All statistical analyses were conducted in GraphPad Prism 10, and the number of animals and cells/animal analyzed is indicated in the corresponding figure legends for this experiment.

#### Astrocyte 2D morphology analysis

High-magnification *Z*-stack images as acquired for NIV analysis were subsequently used to conduct 2D morphology analysis (*n* as described above in astrocyte 3D morphology analysis subsection for P21). Images were flattened in Fiji/ImageJ software through maximum intensity *Z*-projections. Individual brightness levels were adjusted for each cell to enable selection of the complete astrocyte territory with the magic wand selection tool (8-connected mode). Astrocyte morphology measurements included Feret's max and min diameter, aspect ratio, territory area, circularity, and roundness as described in the figure and figure legend for this experiment. The experimenter was blinded to subject group during Fiji/ImageJ analysis. Animal averages were analyzed using appropriate tests (one-way ANOVA with Tukey's post-test or Kruskal–Wallis test with Dunn's multiple-comparisons test). All statistical analyses were conducted in GraphPad Prism 10, and the number of animals and cells/animal analyzed is indicated in the corresponding figure legends for this experiment.

#### Synapse imaging and analysis

Synaptic staining was performed in 25-μm-thick coronal sections containing V1 from P21 *Ptprz1* control (WT and cHet) and cKO mice. Six sex-matched littermate pairs (*n* = 6, three males/three females per group) were collected and used for these experiments. Three different antibody combinations consisting of a presynaptic and postsynaptic target were used to label three different types of synapses: (1) excitatory intracortical, guinea pig anti-VGlut1 (Synaptic Systems 135 304, 1:1,000; RRID: AB_887878)/goat anti-guinea pig IgG Alexa Fluor 647 (Thermo Fisher Scientific A-21240, RRID:AB_2535809) and rabbit anti-PSD95 (Thermo Fisher 51-6900, 1:300; RRID:AB_2533914)/goat anti-rabbit IgG Alexa Fluor 488 (Thermo Fisher Scientific A-11034, RRID:AB_2576217); (2) excitatory thalamocortical, guinea pig anti-VGlut2 (Synaptic Systems 135 404, 1:2,000; RRID:AB_887884)/goat anti-guinea pig IgG Alexa Fluor 647 and rabbit anti-PSD95; and (3) inhibitory, guinea pig anti-VGAT (Synaptic Systems 131 004, 1:1,000; RRID:AB_887873)/goat anti-guinea pig IgG Alexa Fluor 647 and mouse IgG1 anti-gephyrin (Synaptic Systems 147 021, 1:200; RRID:AB_2232546)/goat anti-mouse IgG1 Alexa Fluor 488. Corresponding Alexa Fluor-conjugated secondary antibodies produced in goat were used at 1:200. For VGlut1/PSD95 imaging, high-magnification *z*-stack images containing 15 optical sections spaced 0.34 μm apart were obtained using either a Leica SP8× Falcon (63× objective) or an Olympus FV3000 (60× objective, 1.64× optical zoom) inverted confocal microscope. For VGlut2/PSD95 and VGAT/gephyrin, *z*-stack images containing 15 optical sections spaced 0.34 μm apart were acquired with a 60× objective and 1.64× optical zoom using an Olympus FV3000. Colocalized presynaptic and postsynaptic puncta were quantified using Synbot (Savage et al., 2024) with the following parameters: two channels; noise reduction; manual thresholding; minimum pixel, 3; and pixel overlap. For each staining condition, six sex-matched littermate pairs were collected and analyzed. For each animal, three *z*-stack images (15 slices) were acquired and each image converted in five separate maximum projection images (MPI) of three slices each for a total of 15 MPIs per animal. Animal averages were analyzed using a linear mixed-effects model with genotype and sex as fixed effects. To control for biological variation between litters and technical variation between imaging session, litter and imaging session were included as random effects. The model was fit using REML with the lme4 package (version 1.1-38) in R (version 2025.05.1). The reported *p* value reflects the effect of genotype, and Cohen's *d* with 95% confidence intervals reports the effect size. The individual acquiring the images and performing the analysis was always blinded to the experimental condition. During analysis, one pair for the VGlut1/PSD95 condition was determined to be of suboptimal staining quality and was excluded from analysis. A new pair was added to bring the total *n* to 6.

#### Stimulation emission depletion (STED) microscopy

The 40-µm-thick coronal sections containing V1 from P21 Aldh1l1-EGFP mice were stained with the following antibody combinations: guinea pig anti-VGlut1 (1:1,000) or guinea pig anti-VGlut2 (1:3,000)/goat anti-guinea pig IgG Alexa Fluor 594 (1:100), rabbit anti-PSD95 (1:300)/goat anti-rabbit IgG CF680R (Biotium 20193, 1:100; RRID:AB_10854865), mouse IgG1 anti-phosphacan (clone 3F8, Developmental Studies Hybridoma Bank, University of Iowa; 1:100)/goat anti-mouse IgG1 ATTO 647N (Rockland Immunochemicals 610-156-040, 1:100; RRID: AB_2614870), and chicken anti-GFP (1:1,000)/goat anti-chicken IgY Alexa Fluor 488 (1:100). Three-channel STED images were acquired on the Leica Stellaris 8 FALCON STED using a 100× objective with 2× optical zoom. Channels were acquired in frame sequential mode to minimize cross talk. The white light laser (WLL) allowed for precise selection of excitation wavelength. Software-recommended excitation wavelengths were used for Alexa Fluor 594 and ATTO 647N. The excitation wavelength for CF680R was adjusted to 685 to minimized cross talk with ATTO 647N. A 2D-STED donut was applied, and the 775 nm STED depletion laser was set at 90%. A single confocal channel was acquired simultaneously to visualize GFP-labeled astrocytes. *Z*-stack images were acquired using system-optimized settings for resolution (4,024 × 4,024; image dimension of 58.14 × 58.14 microns) and *z*-stack (seven steps, 0.18 µm). Raw images were exported directly to Huygens Essential for deconvolution.

### Protein extraction and Western blotting

For analysis of cortical lysates via Western blot, P21 *Ptprz1* WT (*n* = 3, two males/one female), cHet (*n* = 3, two males/one female), and cKO (*n* = 3 females) mice were anesthetized with 0.8 mg/kg tribromoethanol (Avertin) and perfused with TBS/heparin to minimize IgG contamination from blood. Immediately following perfusion, brain cortices were rapidly dissected and flash frozen in liquid nitrogen. For brain lysis, one-half of the cortex was homogenized in 1 ml of lysis buffer R (150 mM NaCl, 50 mM Tris, 1 mM EDTA, protease inhibitor mix, 1 mM Na_3_VO_4_, 20 mM NaF, and 10 mM beta-glycerophosphate), pH 7.5, using a ceramic pestle and glass tube. Cortical hemispheres chosen for homogenization were randomized to include both right and left cortices. Homogenate was collected and combined with an equal volume of modified RIPA buffer lacking SDS [M-RIPA: 50 mM Tris, 150 mM NaCl, 1 mM EDTA, 2% NP40, 2% deoxycholate, containing protease inhibitors (Roche 4693132001) 1 mM Na3VO4, 20 mM NaF, and 10 mM beta-glycerophosphate], pH 7.5 . Lysis proceeded for 20 min with rotation at 4°C (12–15 rpm); then the lysate was centrifuged at max speed at 4°C for 10 min. Supernatant was collected, and the Pierce BCA Protein Assay Kit (Thermo Fisher Scientific 23227) was used to determine protein concentration. Samples were stored at −80°C until use.

Chondroitinase ABC lysate digest was performed to remove glycosaminoglycan side chains from PTPRZ1. Chondroitinase ABC (10 U; Sigma-Aldrich C3667-10UN; RRID:AB_2336874) was reconstituted in reconstitution buffer (1% BSA, 50 mM Tris), pH 8.0, and stored at −80°C until use. For the digest, a 2 U/ml chondroitinase ABC working solution was prepared using dilution buffer (2% BSA, 60 mM sodium acetate, 50 mM Tris), pH 8.0, and combined with an equal volume of lysate containing 100 µg. Samples were incubated at 37°C for 90 min and then mixed with 4× Laemmli sample buffer (Bio-Rad Laboratories 1610747) containing 5% β-ME and incubated for 45 min at 45°C for denaturation. A 20 μg of protein was loaded into a 4–15% gradient precast gel (Bio-Rad Laboratories 4561085) or a homemade 6% gel and run at 50 V for 5 min followed by 150 V for 90 min. Proteins were transferred to PVDF membrane (Millipore) at 100 V for 2 h. Immediately following the transfer step, Method 1 provided by the LI-COR Biosciences Revert 700 Total Protein Stain Kit for Western Blot Normalization (Thermo Fisher Scientific NC1145693) was performed to obtain a total protein control stain. Briefly, the PVDF membrane was fully dried, rehydrated in 100% methanol, incubated in Revert 700 Total Protein Stain solution and immediately imaged on a LI-COR Odyssey imaging system. Following total protein detection, the membrane was blocked in intercept blocking buffer (LI-COR Biosciences; VWR 103749-016). Primary antibodies were diluted in 3% BSA in TBS-Tween 20 and incubated in primary antibody overnight at 4°C (rabbit anti-PTPRZ1, 1:1,000, Abcam ab290640). The next day, the membrane was washed three times for 10 min with TBS-Tween 20, incubated in LI-COR secondary antibody solution diluted in Intercept Blocking Buffer (LI-COR IRDye 680RD goat anti-rabbit, 1:5,000; catalog #926-68071, RRID:AB_10956166) for 2 h with agitation at ambient temperature, washed twice with TBS-Tween 20 and once with TBS, dried overnight, and imaged on a LI-COR Odyssey imaging system. Protein expression was quantified using the Image Studio Lite software.

### Quantification and statistical analysis

All statistical analyses were performed in GraphPad Prism 10, with the exception of the Sholl analysis and synapse density experiments where statistical analysis was performed in RStudio as discussed above in the specific subsections. For each experiment, the number of subjects and specific statistical tests are included in the figure legend and data are represented as mean ± standard error of the mean with exact *p* values shown. The effect size is reported as Cohen's *d* for two experimental groups and as eta squared (*η*^2^) for three experimental groups. Sample sizes were determined based on previous experience for each experiment, and no statistical methods were used to predetermine the sample size. Details for inclusion, exclusion, and randomization are included in specific method subsections.

### Data and code availability

All custom code available at https://github.com/BaldwinLabUNC/Astrocyte_morphology. Data are available upon request.

## Results

### PTPRZ1 regulates astrocyte morphogenesis in vitro

**Figure 1.**
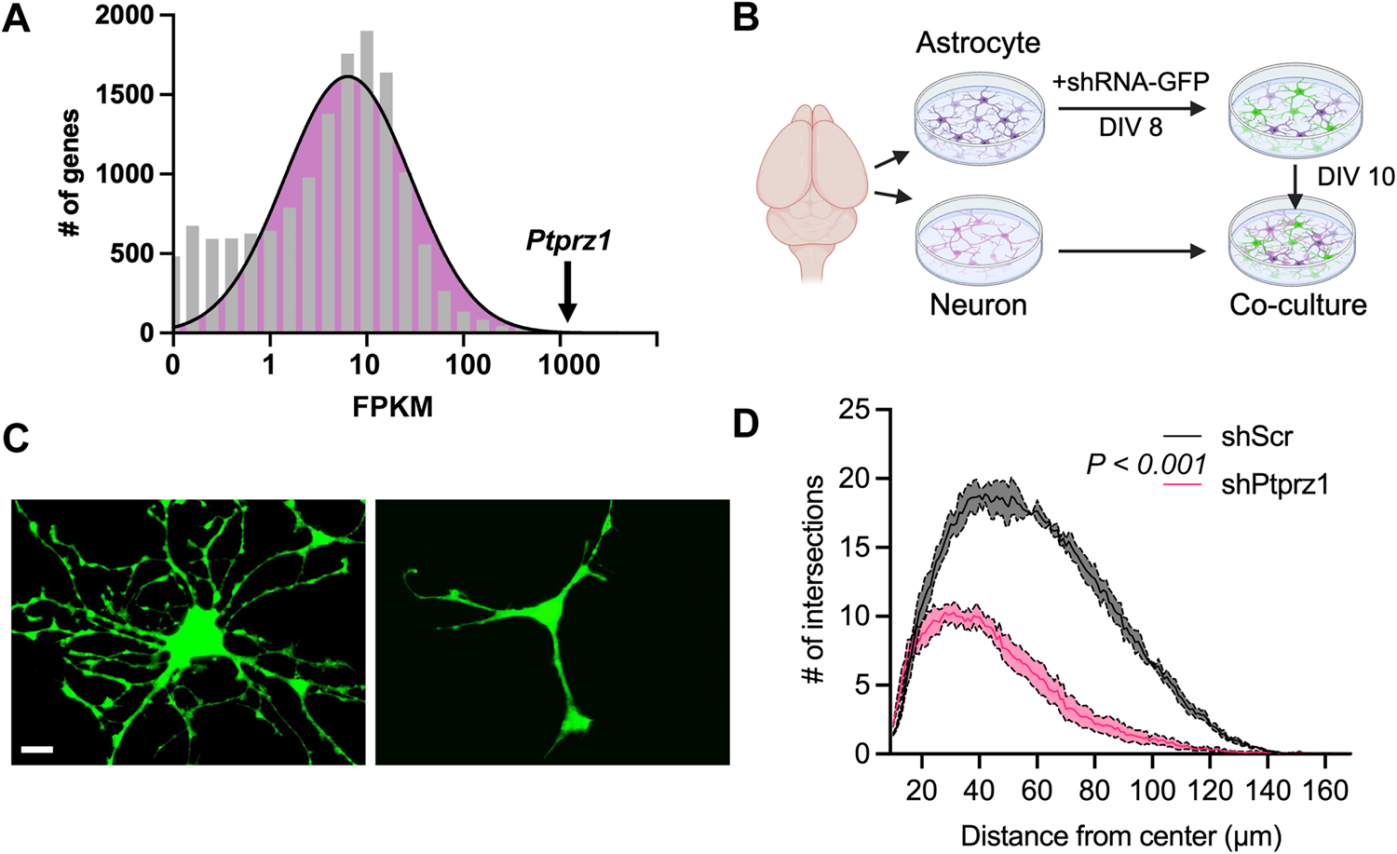
PTPRZ1 regulates astrocyte branching morphogenesis in vitro*.* ***A***, A histogram plot of gene expression levels in purified P7 mouse cortical astrocytes from previously published transcriptomic data (Zhang et al., 2014). *Ptprz1* ranks tenth. ***B***, Workflow for cortical astrocyte–neuron cocultures, created using BioRender.com. ***C***, Representative images of astrocytes cocultured with neurons and expressing scrambled shRNA (shScr) or Ptprz1-targeting shRNA (shPtprz1) and GFP. Scale bar, 10 µm. ***D***, Sholl analysis of astrocyte branching complexity. Solid lines represent mean, and shaded areas represent ±SEM, from three independent experiments, >20 cells/condition/experiment, and linear mixed model with Tukey HSD. Refer to Extended Data Figure 1-1 for information of cell-type–specific expression and shRNA validation.

Previously published transcriptomic studies identified *Ptprz1* as one of the most abundantly expressed genes in astrocytes isolated from the P7 mouse cortex (Fig. 1*A*; Zhang et al., 2014). *Ptprz1* is strongly enriched in astrocytes compared with all other brain cell types (Extended Data Fig. 1-1*A*), and its highest expression levels occur during development, though expression remains high throughout the lifespan (Clarke et al., 2018; Wei et al., 2025). These same expression and enrichment patterns are observed in astrocytes acutely isolated from human brain tissue (Extended Data Fig. 1-1*B*; Zhang et al., 2016). To investigate the function of PTPRZ1 in astrocytes, we first used rat cortical astrocyte and neuron cocultures to determine whether PTPRZ1 is required for astrocyte morphogenesis in vitro. We used short hairpin RNA (shRNA) under control of an hU6 promoter (hU6-shRNA) to knock down *Ptprz1* (shPtprz1; Extended Data Fig. 1-1*C*) in rat cortical astrocytes and confirmed successful protein depletion via Western blot (Extended Data Fig. 1-1*D*). A scrambled shRNA sequence was used as a control (shScr). To visualize the morphology of *Ptprz1*-depleted astrocytes, we generated plasmids expressing both hU6-shRNA and CAG-GFP (Extended Data Fig. 1-1*C*) and transfected these plasmids into rat cortical astrocytes at DIV 8. On DIV 10, transfected astrocytes were cocultured with DIV 10 cortical neurons in serum-free media for 48 h to induce astrocyte ramification. Compared with astrocytes transfected with shScr, shPtprz1 astrocytes showed significantly reduced branching complexity (Fig. 1*C*,*D*), indicating that PTPRZ1 is required for proper astrocyte morphogenesis in vitro.

10.1523/ENEURO.0386-25.2026.f1-1 Figure 1-1 Ptprz1 expression and shRNA validation. **A)** *Ptprz1* gene expression levels per cell type in P7 mouse cortex from Zhang et al., 2014. **B)** PTPRZ1 gene expression levels per cell type in human from Zhang et al., 2016. **C)** Plasmid maps for pLKO.1 vectors used in this study. Plasmids expressing shRNA and puromycin resistance (PuroR) were packaged into lentivirus and transduced into astrocytes to validate shRNA knockdown efficiency (pLKO.1 shRNA-Puro). For morphology analysis, the hPGK promoter and Puro R were replaced with a CAG promoter driving expression of eGFP (pLKO.1 shRNA-GFP). Maps created with BioRender.com. **D)** Western blot of primary rat astrocytes transduced with lentivirus expressing pLKO.1 shScr-Puro or pLKO.1 shPtprz1-Puro and treated with puromycin to eliminate non-transduced astrocytes. PTPRZ1 labeling demonstrates effective knockdown with shPTPRZ1. β-tubulin is used as a loading control. Download Figure 1-1, TIF file.

### Generation of a *Ptprz1* cKO mouse

**Figure 2.**
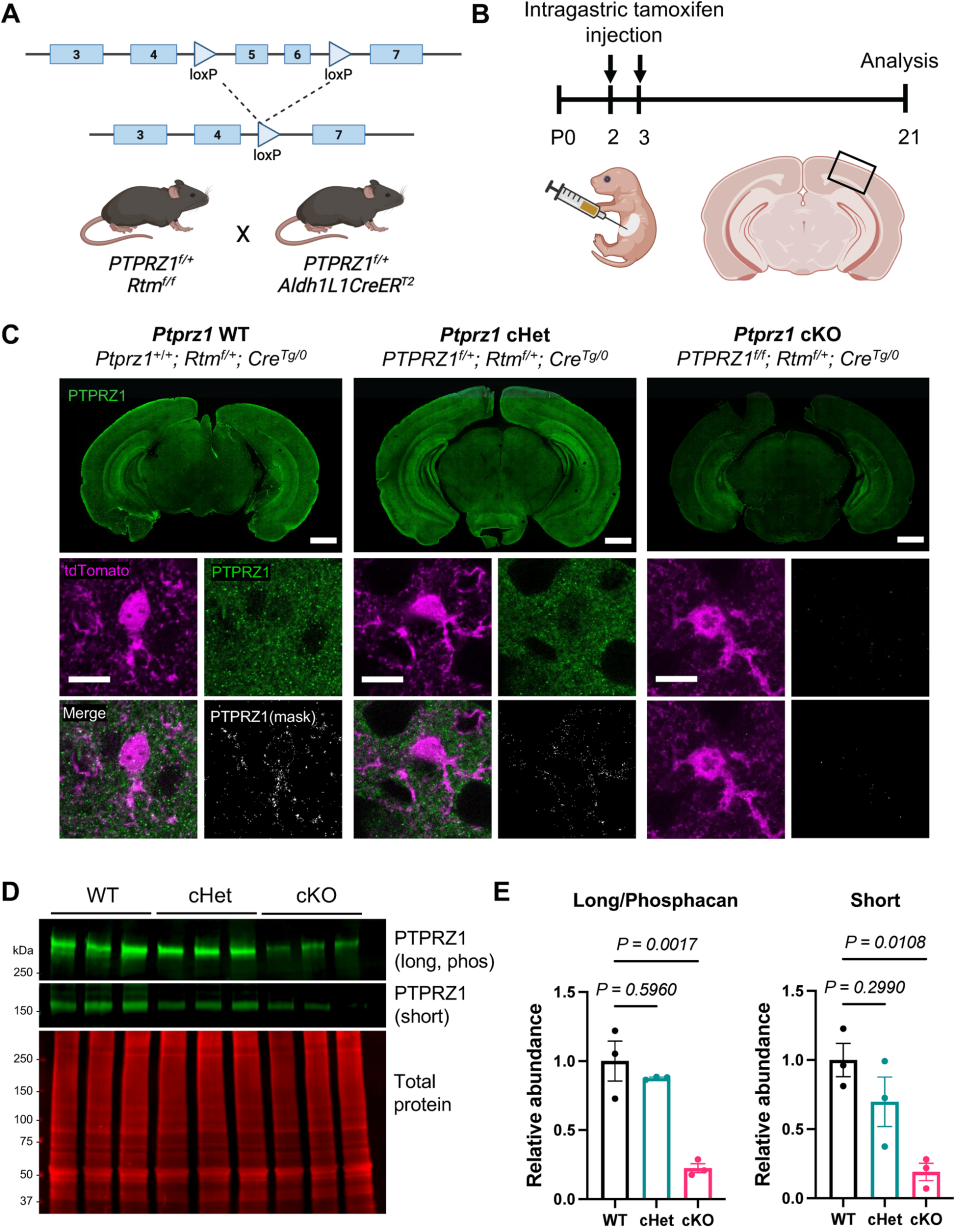
Generation of an astrocyte-specific *Ptprz1* cKO mouse. ***A***, Strategy and breeding scheme for conditional deletion of *Ptprz1* from astrocytes, created using BioRender.com. ***B***, Experimental timeline for intragastric tamoxifen administration and tissue collection and analysis of the visual cortex, created using BioRender.com. ***C***, Representative tile scan images of P21 coronal sections containing the primary visual cortex (V1; top) and high-magnification images of individual V1 L5 astrocytes (Td-Tomato; bottom) from *Ptprz1* WT, *Ptprz1* cHet, and *Ptprz1* cKO mice. Immunolabeling for PTPRZ1 (green) and Td-Tomato (magenta) demonstrates effective deletion of PTPRZ1 from astrocytes. Binarized masks show PTPRZ1 localization within astrocyte cell bodies and processes. Overview scale bar, 1,000 µm; high-magnification scale bar, 10 µm. ***D***, Western blot image of cortical lysates from three WT, three cHet, and three cKO mice at P21. Lysates were digested with chondroitinase ABC to detect all three isoforms of PTPRZ1 with total protein as a loading control. ***E***, Quantification of fluorescent band intensity of PTPRZ1 long and phosphacan and PTPRZ1 short normalized to total protein. Data are presented as mean ± SEM; *n* = 3 mice per genotype. One-way ANOVA, Tukey's post-test. Effect size: long *η*^2^ = 0.8876; short *η*^2^ = 0.7678. Refer to Extended Data Figure 2-1 and Extended Data Figure 2-2 for additional validation experiments.

Previous studies observed that in vitro astrocyte morphology phenotypes often manifest differently in vivo (Stogsdill et al., 2017; Baldwin et al., 2021), likely due to the numerous differences between in vitro and in vivo microenvironments. Thus, to determine the function of PTPRZ1 in astrocytes during brain development, we generated a transgenic mouse line to conditionally delete *Ptprz1* from astrocytes. To do so, we used a BAC with homology to the *Ptprz1* locus to insert *loxP* sites flanking exons 5 and 6 (Fig. 2*A*,*B*). Cre-mediated excision of Exons 5 and 6 introduces a premature stop codon in the N-terminal carbonic anhydrase domain that is present in all three PTPRZ1 isoforms.

10.1523/ENEURO.0386-25.2026.f2-1 Figure 2-1 Validation of PTPRZ1 expression in OPCs and neurons following conditional *Ptprz1* deletion in astrocytes. **A-B)** High-magnification images of individual V1 L5 **(A)** and corpus callosum (CC); **B**) oligodendrocyte precursor cells (OPCs; PDGFRα) from *Ptprz1* WT (left) and *Ptprz1* cKO (right) mice. Immunolabeling for PDGFRα (magenta) and PTPRZ1 (green) demonstrates high PTPRZ1 expression by OPCs independent of genotype. Binarized masks show PTPRZ1 localization within OPC cell bodies and processes. Scale bars = 10μm. **C-D)** High-magnification images of neuronal cell bodies and processes (β3-Tubulin) in V1 L5 **(C)** and CC **(D)** from *Ptprz1* WT (left) and *Ptprz1* cKO (right) mice. Immunolabeling for β3-Tubulin (magenta) and PTPRZ1 (green) demonstrates low PTPRZ1 expression by neurons independent of genotype. Binarized masks show PTPRZ1 localization within neuronal cell bodies and proximal processes **(C)** or axon tracts **(D)**. Scale bars = 10μm. Download Figure 2-1, TIF file.

10.1523/ENEURO.0386-25.2026.f2-2 Figure 2-2 Additional validation of *Ptprz1* conditional deletion. **A)** Genotyping strategy for detecting wild-type (WT) and floxed *Ptprz1* alleles from mouse genomic DNA. The same forward and reverse primers detect both alleles, with the floxed allele appearing 89 base pairs higher due to the addition of the *loxP* site. **B)** Example of PCR products obtained from WT (+/+), f/+, and f/f mice. **C)** Western blot of PTPRZ1 showed failed separation of long and secreted isoforms on a 6% gel. **D)** Representative tile scan images of P7 coronal sections containing primary VCX (top) and high-magnification images of individual V1 L5 astrocytes (Td-Tomato; bottom) from *Ptprz1* WT and *Ptprz1* cKO mice. Immunolabeling for PTPRZ1 (green) and Td-Tomato (magenta) demonstrates effective deletion of PTPRZ1 from astrocytes. Binarized masks show PTPRZ1 localization within astrocyte cell bodies and processes. Overview scale bar 1000 µm, high-magnification scale bar 10 µm. Download Figure 2-2, TIF file.

To delete *Ptprz1* specifically from astrocytes during early postnatal development, we crossed *Ptprz1* flox mice with Aldh1L1CreER^T2^ transgenic mice and administered tamoxifen intragastrically at P2 and P3 (Fig. 2*B*). We chose this tamoxifen administration timepoint for three reasons. First, astrocyte-specific deletion with this transgene is unsuccessful prior to astrogenesis, and the majority of astrogenesis in the cortex is complete by P2 (Ge et al., 2012). Second, PTPRZ1 expression is associated with stemness and pluripotency in human outer RGCs (Pollen et al., 2015) and suppression of PTPRZ1 promotes OPC differentiation (McClain et al., 2012). Whether PTPRZ1 is involved in astrogenesis is unknown and is complicated by our lack of understanding of the early stages of astrocyte maturation. Deleting *Ptprz1* at the conclusion of astrogenesis eliminates this potentially confounding variable and allows study of astrocyte maturation. Third, studies have previously demonstrated that this tamoxifen administration strategy expresses Cre in astrocytes with high specificity and efficiency (Baldwin et al., 2021). To control for any unanticipated phenotypes associated with transgene expression, all mice used in this study expressed exactly one copy of the Cre transgene. These mice also expressed one allele of the Rosa td-Tomato Cre reporter transgene to visualize Cre-expressing cells.

To confirm successful deletion of PTPRZ1 in astrocytes, we performed immunolabeling of brain tissue sections with a phosphacan antibody (3F8) that detects both the secreted and long isoforms of PTPRZ1. At P21, we observed a substantial decrease in PTPRZ1 protein expression in gray matter brain regions of *Ptprz1* cKO mice (*Ptprz1^f/f^*, *RTM^f/+^*, *Cre^Tg/0^*), but not in *Ptprz1* conditional heterozygous (cHet) mice (*Ptprz1^f/+^*, *RTM^f/+^*, *Cre^Tg/0^*), compared with WT (*Ptprz1^+/+^*, *RTM^f/+^*, *Cre^Tg/0^*; Fig. 2*C*). This deletion was specific to astrocytes, as PTPRZ1 labeling was absent in td-Tomato^+^ astrocytes of *Ptprz1* cKO mice (Fig. 2*C*) yet present in PDGFRα^+^ OPCs (Extended Data Fig. 2-1*A*,*B*) and β3-tubulin^+^ neurons (Extended Data Fig. 2-1*C*,*D*). To quantitatively assess the reduction in PTPRZ1 protein levels, we performed Western blot of cortical lysates from *Ptprz1* WT, cHet, and cKO mice at P21. Following digest with chondroitinase ABC, all three isoforms were detectable by Western blot, though the bands for the long isoform and phosphacan could not be adequately separated for quantification, consistent with prior studies (Nishiwaki et al., 1998; Fig. 2*D*; Extended Data Fig. 2-2*C*). We observed a 78% reduction in PTPRZ1-long/phosphacan expression in cKO compared with WT mice, as well as an 81% reduction in PTPRZ1-short expression (Fig. 2*E*). These results indicate that a majority of PTPRZ1 expression at P21 (∼80%) is derived from astrocytes, while the remaining 20% is derived from other PTPRZ1-expressing cells, including OPCs and neurons. To confirm successful deletion prior to astrocyte morphogenesis and synaptogenesis, we performed immunolabeling of the P7 mouse cortex with 3F8/phosphacan and confirmed a substantial decrease in PTPRZ1 protein in cKO mice compared with WT (Extended Data Fig. 2-2*D*). Collectively, these results demonstrate efficient deletion of *Ptprz1* from astrocytes in the mouse cortex during development.

### Postnatal astrocyte-specific deletion of *Ptprz1* does not alter cell number at P21

**Figure 3.**
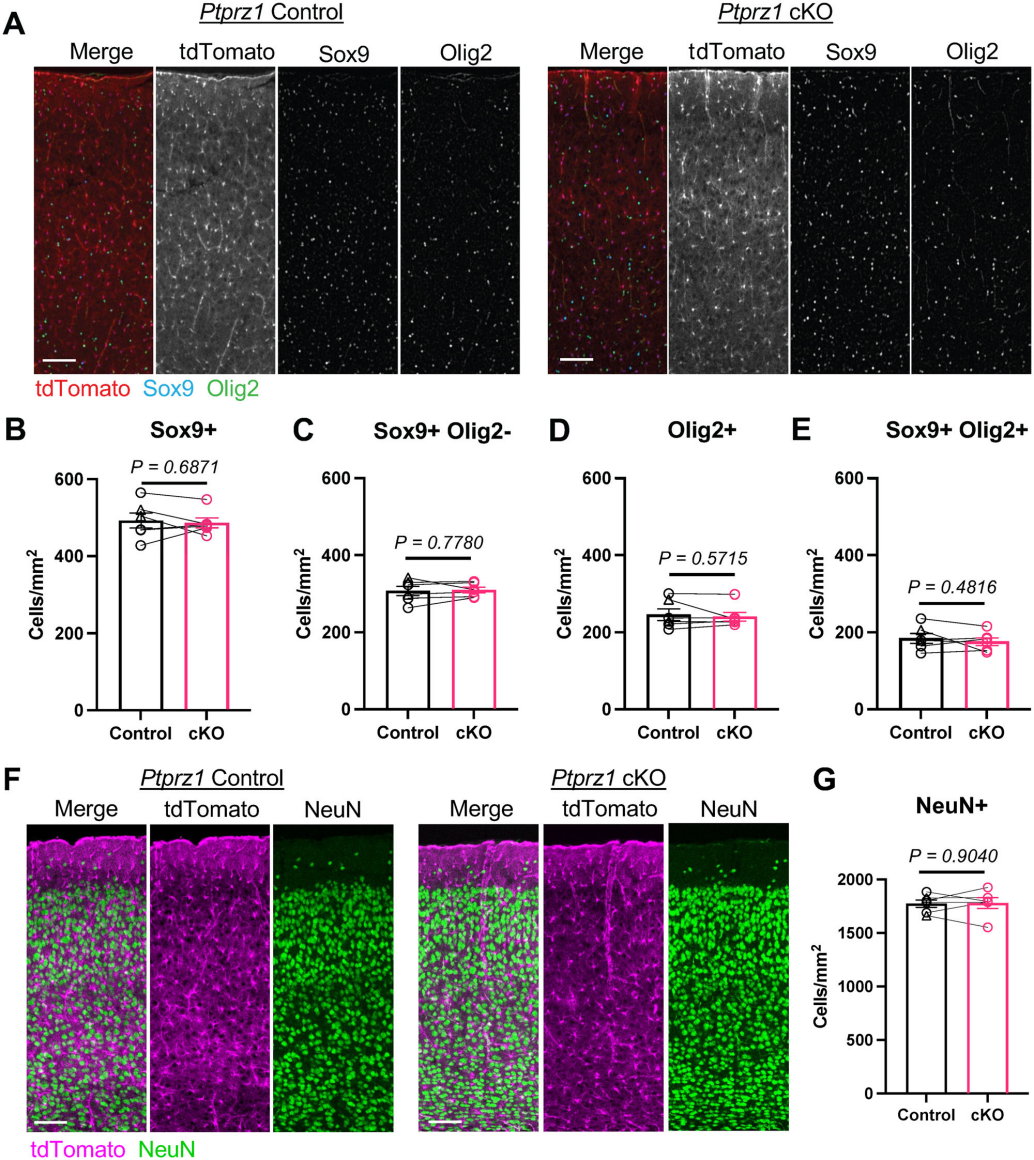
Postnatal deletion of *Ptprz1* from astrocytes does not impact cortical cell numbers at P21. ***A***, Representative merged and single-channel tile scan images of the P21 V1 from *Ptprz1* control (WT and cHet) and *Ptprz1* cKO mice with tdTomato^+^ (red) astrocytes and immunolabeling for Sox9 (blue) and Olig2 (green). Scale bar, 100 µm. ***B–E***, Quantification of the number of cells from panel ***A*** images for (***B***) Sox9^+^, *d* = 0.246, CI [−1.237, 1.730]; (***C***) Sox9^+^/Olig2^−^ (astrocytes), *d* = −0.172, CI [−1.656, 1.312]; (***D***) Olig2^+^ (oligodendrocyte-lineage cells), *d* = 0.349, CI [−1.135, 1.833]; and (***E***) Sox9^+^/Olig2^+^, *d* = 0.439, CI [−1.045, 1.923] nuclei. *n* = 6 sex-matched littermate pairs of control and cKO mice (3 male, 3 female). Bar graphs show mean ± SEM. Lines connect sex-matched control–cKO littermate pairs. Data points represent per animal averages of three images. In the control column, a triangle denotes WT mice and circle denotes cHet. *p* values were calculated using a linear mixed-effects model. Effect size reported above as Cohen's *d* with 95% confidence intervals (CI [lower, upper]). ***F***, Representative merged and single-channel tile scan images of the P21 V1 from *Ptprz1* control (WT and cHet) and *Ptprz1* cKO mice with tdTomato^+^ (magenta) astrocytes and immunolabeling for NeuN (green). Scale bar, 100 µm. ***G***, Quantification of cells from panel ***F*** images with NeuN^+^ (neurons) *d* = −0.073, CI [−1.577, 1.411] nuclei from *n* = 6 sex-matched littermate pairs of control and cKO mice. Bar graphs show mean ± SEM. Lines connect sex-matched control–cKO littermate pairs (3 male, 3 female). Data points represent per animal averages of three images. *p* value and effect size as in ***B–E***. Refer to Extended Data Figure 3-1 for information on the image analysis workflow.

Astrocytes undergo local division following differentiation in late embryonic and early postnatal stages. A previous study found that at P3, 18.9% of cortical astrocytes were in the process of cell division, and this number decreased to 13.1% at P6 and 1.5% at P14 (Ge et al., 2012). Following tamoxifen administration at P2 and P3, we observed astrocyte-specific deletion of PTPRZ1 by P7, near the end of postnatal astrocyte proliferation (Extended Data Fig. 2-2*D*). Because PTPRZ1 is expressed in different stem cell populations and may play roles in cell proliferation and/or differentiation, we first examined whether postnatal deletion of *Ptprz1* from astrocytes impacted total astrocyte number at P21, a timepoint by which astrocytes are morphologically mature (Stogsdill et al., 2017; Baldwin et al., 2021). For quantification, we developed a new semiautomated workflow (Extended Data Fig. 3-1*A*–*D*) based on a published nuclear labeling strategy (Baldwin et al., 2021; Fig. 3*A*). We focused our studies on the primary visual cortex (V1), where sparse labeling strategies are known to be effective, layer-specific effects can be readily assessed, sensory input can be easily controlled, and astrocyte biology has been broadly studied (Blanco-Suarez et al., 2018; Baldwin et al., 2021; Lee et al., 2025). Because we did not observe significant differences in protein expression between WT and cHet mice (Fig. 2*C*–*E*), we combined these two genotypes for our control group for these experiments. At P21, we found no difference in the number of astrocytes (Sox9^+^/Olig2^−^) between *Ptprz1* cKO mice and sex-matched littermate controls (Fig. 3*B*,*C*). Neuron (NeuN^+^) and oligodendrocyte-lineage cell (Olig2^+^) numbers were also unaffected (Fig. 3*D*–*G*). These results demonstrate that early postnatal deletion of *Ptprz1* from astrocytes does not impact the overall number of cortical astrocytes, neurons, or oligodendrocyte-lineage cells at P21.

10.1523/ENEURO.0386-25.2026.f3-1 Figure 3-1 Cell counting workflow. **A)** Representative input image for the cell count pipeline. Cropped grayscale image of visual cortex with Sox9-labeled nuclei. Green arrows denote representative Sox9 + nuclei that will eventually be included in the cell count. Magenta arrows denote representative debris that will eventually be excluded from the cell count. **B)** Processed input image, after denoising, foreground signal enhancement, and nuclei-specific signal enhancement. Signal separates more clearly from the background, and nuclei appear more distinct from debris, compared to the original image. **C)** Identified objects segmented from the processed image. Both nuclei and debris are identified as objects. **D)** Objects representing debris are filtered out. Objects representing Sox9 + nuclei remain and are included in the quantification of Sox9 + cells in the image. Download Figure 3-1, TIF file.

### Astrocyte-specific *Ptprz1* deletion has minimal impact to astrocyte morphology at P21

**Figure 4.**
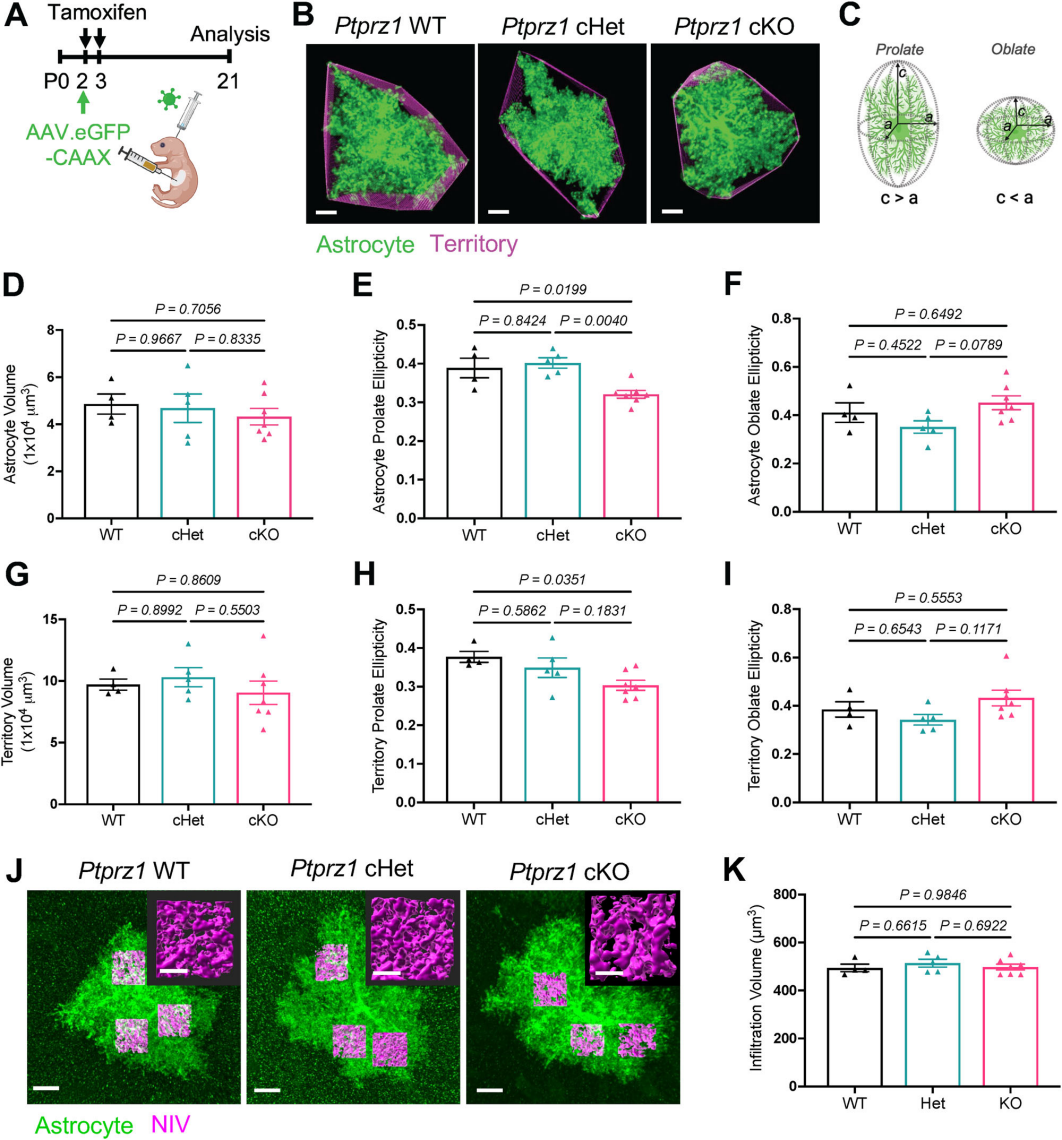
*Ptprz1* cKO astrocytes display altered morphology at P21. ***A***, Tamoxifen administration and AAV injection strategy for *Ptprz1* cKO and sparse labeling for detailed morphology analyses. ***B***, V1 L5 astrocytes at P21 expressing eGFP-CAAX (green) in *Ptprz1* WT (left), *Ptprz1* cHet (center), and *Ptprz1* cKO (right) mice. Astrocyte territory in magenta. Scale bar, 10 µm. ***C***, Schematic describing prolate versus oblate ellipticity as a difference in whether the ellipse is rotated about a major (longest) axis or minor (shortest) axis. Created using BioRender.com. ***D–I***, 3D morphology analyses of (***D***) astrocyte volume (*η*^2^ = 0.0534), (***E***) astrocyte prolate ellipticity (*η*^2^ = 0.594), (***F***) astrocyte oblate ellipticity (*η*^2^ = 0.304), (***G***) territory volume (*η*^2^ = 0.0814), (***H***) territory prolate ellipticity (*η*^2^ = 0.402), and (***I***) territory oblate ellipticity (*η*^2^ = 0.266) in P21 V1 L5 astrocytes. Data presented as subject averages [individual mice represented as triangles; *n* = 4 (WT), *n* = 5 (cHet), and *n* = 7 (cKO) mice per group, 4–8 cells per mouse]. Bars are mean ± SEM. One-way ANOVA, Tukey’s post-test. Effect size reported as *η*^2^. ***J***, Representative V1 L5 astrocytes at P21 expressing eGFP-CAAX (green) with NIV reconstructions (magenta; inset scale, 5 µm). Scale bar, 10 μm. ***K***, NIV analysis for *Ptprz1* WT, cHet, and cKO astrocytes (*η*^2^ = 0.0695). Three ROIs/cell, 4–5 cells/mouse, *n* as in ***I***. Data presented as subject averages as above (***D–I***). Bars are mean +/− SEM. One-way ANOVA, Tukey's post-test. Effect size reported as *η*^2^. Refer to Extended Data Figures 4-1, 4-2, and 4-3 for additional analysis of astrocyte morphology.

To determine whether *Ptprz1* is necessary for astrocyte morphogenesis in vivo, we performed a comprehensive assessment of astrocyte morphology in V1 of *Ptprz1* WT, cHet, and cKO mice at P21, a timepoint by which the bulk of astrocyte morphogenesis has occurred. To accurately capture the morphology of individual cells, we performed unilateral intracortical injection of PHP.eB serotype AAV at P2 to sparsely label astrocytes with membrane-targeted GFP (GFP-CAAX) under control of the human minimal GFAP promoter (gfaABC1D; Fig. 4*A*). We acquired confocal *z*-stack images of entire astrocyte volumes from individual V1 L5 astrocytes and analyzed 3D astrocyte architecture using a published workflow (Eaker and Baldwin, 2022; Fig. 4*B*,*C*). While we observed robust expression of PTPRZ1 in all layers of the cortex (Fig. 2*C*), we chose to examine astrocyte morphology in V1 L5 due to the efficiency of this viral approach in targeting deeper layer astrocytes and the large body of literature characterizing V1 L5 astrocyte morphology. We performed statistical analysis both on a per-animal (Fig. 4*D*–*I*) and per-cell (Extended Data 4-1*A*–*J*) basis. We base all of our conclusions on per animal analysis but include per-cell analysis for additional transparency as this reveals any variability within a larger population of cells and is currently the more common presentation format in the field (Endo et al., 2022; Cheng et al., 2023; Rosenberg et al., 2023; Szewczyk et al., 2024; Lee et al., 2025).

10.1523/ENEURO.0386-25.2026.f4-1 Figure 4-1 Additional 3D morphological analyses of V1 L1 and L5 astrocytes. **(A-F)** Individual astrocyte statistics represented by open circles for 3D morphology metrics presented in Fig. 4: **(A)** astrocyte volume, **(B)** astrocyte prolate ellipticity, **(C)** astrocyte oblate ellipticity, **(D)** territory volume, **(E)** territory prolate ellipticity, and **(F)** territory oblate ellipticity in P21 V1 L5 astrocytes. For each metric, n = 4 (WT), n = 5 (cHet) and n = 7 (cKO) mice per group, 4-8 cells per mouse. Bars are mean +/- SEM. One-way ANOVA, Tukey’s post-test. **(G-J)** Additional 3D morphology analyses of: **(G)** Analysis of territory area (η^2^ = 0.079), **(H)** territory sphericity, **(I)** astrocyte surface area (η^2^ = 0.0404), and **(J)** astrocyte sphericity (η^2^ = 0.0191) in P21 V1 L5 astrocytes. Data presented as subject averages (individual mice represented as triangles; n as in **(A-F)**, 4-8 cells per mouse). Bars are mean +/- SEM. One-way ANOVA, Tukey’s post-test, effect size reported as η^2^ (G, I-J) or Kruskal-Wallis test, Dunn’s multiple comparisons test (H). **K)** NIV analysis as in Fig. 4** K** for individual astrocytes, represented by open circles. Three ROIs/cell, 4-5 cells/mouse, n as in **(A-F)**. Bars are mean +/- SEM. One-way ANOVA, Tukey’s post-test. **L)** Representative V1 L1 astrocytes at P21 expressing eGFP-CAAX (green) with Neuropil Infiltration Volume (NIV) reconstructions (magenta; inset scale = 5 µm). Scale bar 10μm. **M)** NIV analysis for *Ptprz1* control (WT + cHet) and cKO astrocytes. Open circles represent individual astrocytes from 11 animals, n = 25 cells per group. Bars are mean +/- SEM. Unpaired t-test. Download Figure 4-1, TIF file.

10.1523/ENEURO.0386-25.2026.f4-2 Figure 4-2 Additional 2D morphological analyses of V1 L5 astrocytes. **(A-F)** 2D morphology analyses of: **(A)** Feret’s max diameter (η^2^ = 0.0799), **(B)** Feret’s min diameter, **(C)** aspect ratio (Feret Max/Feret Min; η^2^ = 0.0859), **(D)** territory area, **(E)** circularity (η^2^ = 0.488), and **(F)** roundness (η^2^ = 0.0783). Triangles represent subject averages (n = 4 (WT), n = 5 (cHet) and n = 7 (cKO) mice per group, 4-6 cells per mouse). Bars are mean +/- SEM. One-way ANOVA, Tukey’s post-test, effect size reported as η^2^ (A, C, E-F) or Kruskal-Wallis test, Dunn’s multiple comparisons test (B, D). Download Figure 4-2, TIF file.

10.1523/ENEURO.0386-25.2026.f4-3 Figure 4-3 Modest morphology differences at P14 in *Ptprz1* cHet. **A)** Tamoxifen administration and AAV injection strategy for *Ptprz1* cKO and sparse labeling for detailed morphology analyses. **B)** V1 L5 astrocytes at P14 expressing mCherry-CAAX in *Ptprz1* WT (left), *Ptprz1* cHet (center) and *Ptprz1* cKO (right) mice. Astrocytes expressing mCherry-CAAX in green; astrocyte territory in magenta. Scale bar, 10μm. **(C-L)** 3D morphology analyses of: **(C)** astrocyte sphericity (η^2^ = 0.258), **(D)** astrocyte volume (η^2^ = 0.0606), **(E)** astrocyte prolate ellipticity (η^2^ = 0.128), **(F)** astrocyte oblate ellipticity (η^2^ = 0.0426), **(G)** territory sphericity (η^2^ = 0.202), **(H)** territory volume (η^2^ = 0.112), **(I)** territory prolate ellipticity (η^2^ = 0.0658), **(J)** territory oblate ellipticity (η^2^ = 0.0248), **(K)** astrocyte surface area (η^2^ = 0.183), and **(L)** territory area (η^2^ = 0.121) in P14 V1 L5 astrocytes. Data presented as subject averages (individual mice represented as triangles; n = 8 mice per group, 5 cells per mouse). Bars are mean +/- SEM. One-way ANOVA, Tukey’s post-test. Effect size reported as η^2^. **M)** Representative V1 L5 astrocytes at P14 expressing mCherry-CAAX (green) with Neuropil Infiltration Volume (NIV) reconstructions (magenta; inset scale = 5μm). Scale bar 10μm. (**N)** NIV analysis for *Ptprz1* WT, cHet and cKO astrocytes (η^2^ = 0.285). Three ROIs/cell, 5 cells/mouse, n as in **(C-L)**. **N)** Data presented as subject averages as above **(C-L)**. Bars are mean +/- SEM. One-way ANOVA, Tukey’s post-test. Effect size reported as η^2^. Download Figure 4-3, TIF file.

Though we observed a dramatic reduction of morphological complexity upon *Ptprz1* knockdown in vitro, we did not observe substantial differences in astrocyte morphological complexity at P21 between WT, cHet, and cKO mice. We analyzed both astrocyte surfaces and astrocyte convex hulls generated in Imaris for a number of metrics, including total volume, sphericity, ellipticity, and surface area (Fig. 4*D*–*I*; Extended Data Fig. 4-1*A*–*J*). We found no significant differences in volume, sphericity, or surface area between any of the genotypes. We did, however, observe significant differences in ellipticity of both astrocyte territories and astrocyte cell volumes, with *Ptprz1* cKO astrocytes having a lower prolate ellipticity index (Fig. 4*E*,*H*) compared with controls. This finding indicates that *Ptprz1* cKO astrocytes are less elongated (prolate) relative to WT or cHet astrocytes at P21. Note that ellipticity is measured independently of anatomical orientation and is based on the intrinsic shape of the cell (Fig. 4*C*).

To determine whether PTPRZ1 is required for astrocytes to form finer branches that infiltrate the neuropil, we acquired high-resolution images from thinner (40 µm) sections and analyzed 3D regions of interest within labeled V1 L5 astrocyte territories to quantify NIV. Astrocytes are a heterogeneous cell population, and astrocytes in different cortical layers exhibit distinct molecular features, particularly in L1, a layer that is rich in synapses, sparse in neuronal cell bodies, and adjacent to the pia (Bayraktar et al., 2020; Bocchi et al., 2025). Because we observed strong PTPRZ1 expression in L1, we also examined astrocyte NIV in this layer. However, because our labeling approach only sparsely labels upper layer astrocytes, we did not obtain sufficient labeling to perform 3D volume analysis in L1. In contrast to our in vitro findings, loss of *Ptprz1* did not impact astrocyte neuropil infiltration in V1 L5 or L1, at least at the level of confocal resolution (Fig. 4*J*,*K*; Extended Data Fig. 4-1*L*,*M*). We also performed an additional multipoint 2D analysis on maximum projections of V1 L5 astrocytes using methods developed in a previous study (Endo et al., 2022). We observed no significant differences in any of the measurements aside from an increase in circularity in cHet astrocytes (Extended Data Fig. 4-2*A*–*F*). Because this difference was not reflected in the 3D morphology measurements, which capture cell geometry in more detail, we interpret this as an artifact of the 2D maximum projection process. Collectively, these results demonstrate the modest role of astrocytic PTPRZ1 in regulating astrocyte geometry at P21.

Given that PTPRZ1 expression in the mouse cortex is highest at P7 (Wei et al., 2025), we reasoned that earlier developmental timepoints may show a more pronounced morphological phenotype that resolves by P21. Therefore, we performed the same battery of morphological tests with V1 L5 astrocytes at P14 (Extended Data Fig. 4-3*A*,*B*). We found no significant differences in cell volume, territory volume, surface area, or ellipticity at this time point (Extended Data Fig. 4-3*C*–*L*), though we did observe significantly increased sphericity for cHet cells (Extended Data Fig. 4-3*F*). We also observed a small, yet significant increase in neuropil infiltration for cHet astrocytes at P14 (Extended Data Fig. 4-3*M*,*N*) that is resolved by P21 (Fig. 4*K*). Across all P14 metrics, we did not observe any significant differences in WT and cKO comparisons or cHet and cKO comparisons.

### Detection of PTPRZ1 at tripartite synapses

In the developing mouse cortex, astrocytes closely associate with neuronal synapses and promote synapse formation and maturation via both secreted factors and direct contact (Blanco-Suarez et al., 2018; Irala et al., 2024; Bosworth et al., 2025). PTPRZ1 expression has been detected at excitatory synapses in cultured neurons and in the adult rat hippocampus (Hayashi et al., 2005a; Dino et al., 2006). Behavioral studies reveal impaired spatial learning (Niisato et al., 2005) and contextual fear memory (Tamura et al., 2006) in constitutive *Ptprz1* KO mice, indicative of synaptic deficit. Together, these findings suggest that PTPRZ1 could play an important role at the synapse; however, the function of PTPRZ1 at the synapse during development is unknown.

**Figure 5.**
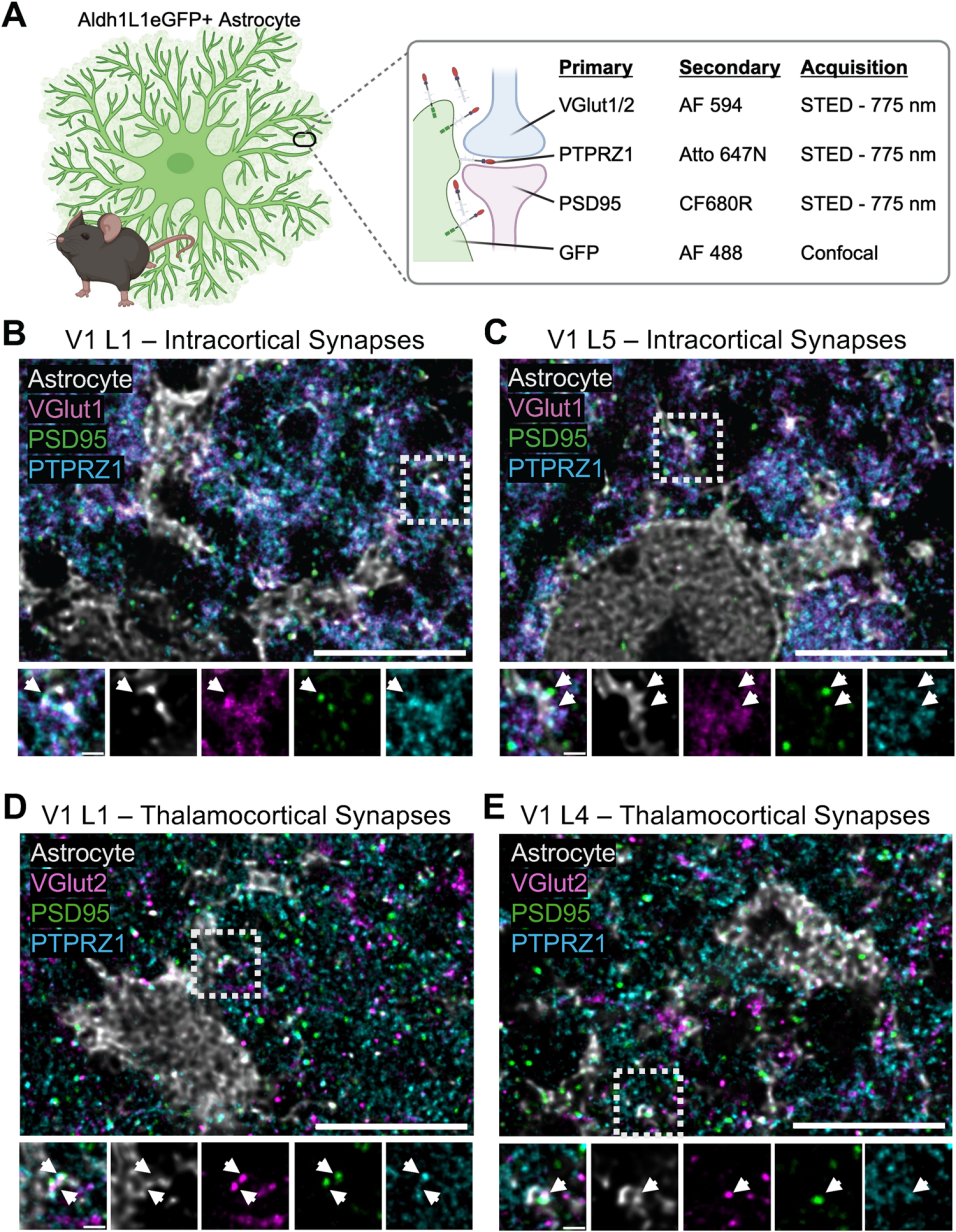
Super-resolution STED microscopy reveals PTPRZ1 localization to excitatory synapses. ***A***, Schematic of experiment workflow created using BioRender.com. AF, Alexa Fluor. ***B***, Super-resolution STED image of PTPRZ1 (cyan) localized to excitatory synapses identified by close proximity of PSD95 (green, postsynaptic marker) and VGlut1 (magenta; intracortical presynaptic marker) in close proximity to an astrocyte process (gray, acquired with confocal imaging). Top, distribution of PTPRZ1, PSD95, and VGlut1 in P21 V1 L1; scale bar, 5 μm; white box indicates ROI shown below. Bottom, Close proximity of PTPRZ1 to an excitatory synapse (PSD95/VGlut1) contacted by an astrocyte process; scale bar, 0.5 μm. ***C***, As in ***B***, for V1 L5. ***D–E***, As in ***B***, VGlut2 (magenta; thalamocortical presynapse) replaces VGlut1 as a presynaptic excitatory synapse marker for (***D***) V1 L1 and (***E***) V1 L4.

To further investigate the function of astrocyte-derived PTPRZ1, we examined its subcellular localization and proximity to synapses in the P21 mouse V1. To do so, we designed a workflow for detecting protein expression at tripartite synapses by multiplexing confocal imaging of fluorescently labeled astrocytes and three-color stimulated emission depletion (STED) with a single 775 nm depletion laser (Fig. 5*A*). We performed immunolabeling of tissue sections from Aldh1L1-eGFP mice with antibodies to detect PTPRZ1, GFP (astrocytes), PSD95 (excitatory post synapse), and either VGlut1 to detect excitatory intracortical synapses or VGlut2 to detect excitatory thalamocortical synapses. Using the Leica Stellaris FALCON 8 STED equipped with a WLL, we acquired four-channel images with the following combination of confocal and STED channels: (1) STED, VGlut1 or VGlut2, Alexa Fluor 594; (2) STED, PTPRZ1, ATTO 647N; (3) STED, PSD95, CF680R; and (4) confocal, GFP, Alexa Fluor 488 (Fig. 5*A*). We focused on V1 L1 and L5 excitatory intracortical synapses due to the strong expression of PTPRZ1 in L1, the bulk of our morphology analyses being conducted in L5, and the abundance of this synapse type in these layers. We examined excitatory thalamocortical synapses in V1 L1 and L4 due to the preferential targeting of excitatory thalamocortical inputs specifically to these layers (Kloc and Maffei, 2014). Using this setup, we observed PTPRZ1 expression along astrocyte branches and at synapses. PTPRZ1 expression colocalized with GFP^+^ astrocyte processes at excitatory intracortical synapses in V1 L1 and L5 (Fig. 5*B*,*C*) and excitatory thalamocortical synapses in V1 L1 and L4 (Fig. 5*D*,*E*).

### Astrocyte-derived PTPRZ1 regulates the density of colocalized excitatory synapse markers

**Figure 6.**
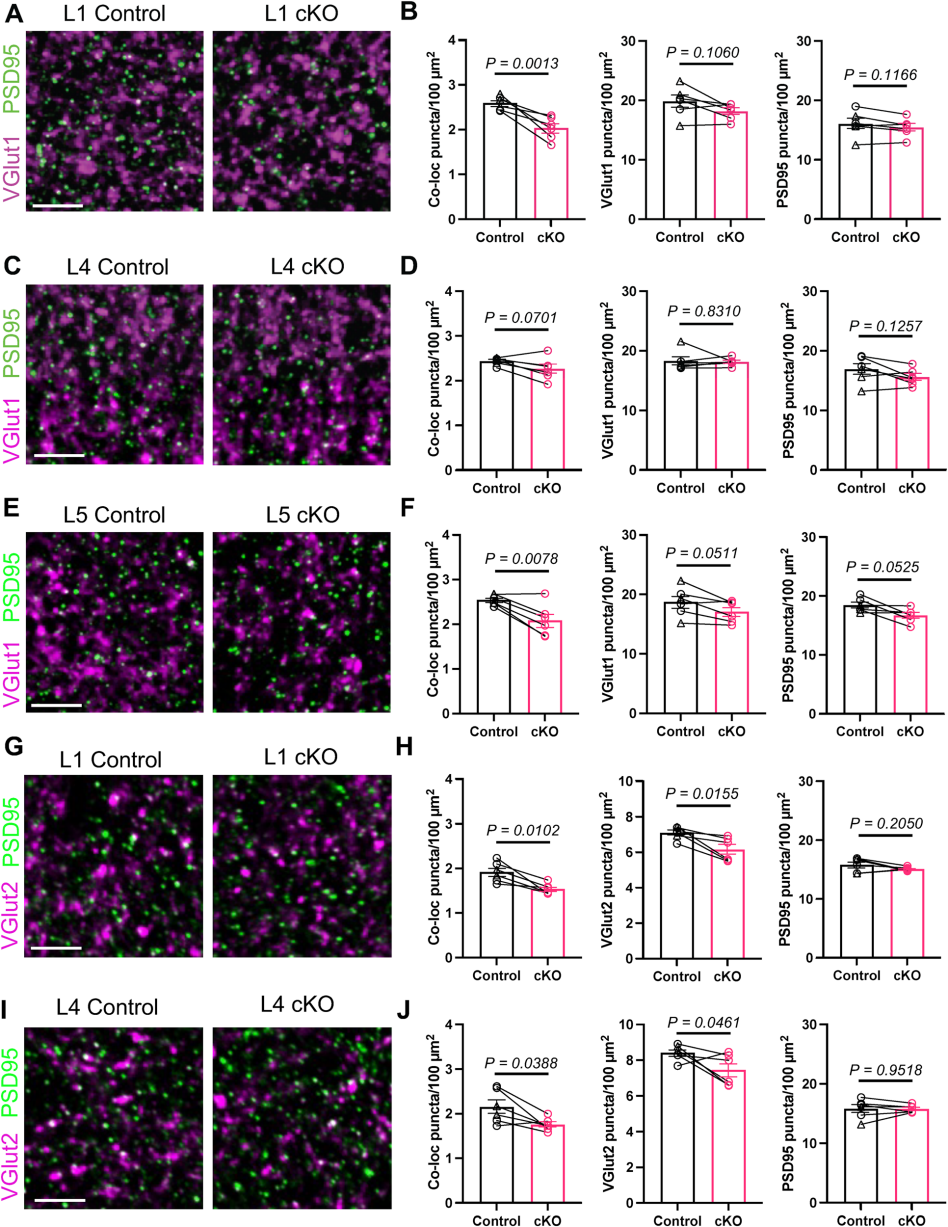
Astrocytic PTPRZ1 regulates the density of colocalized excitatory synapse markers in vivo at P21. ***A***, Representative 17.5 × 17.5 µm ROIs labeled with excitatory intracortical synapse markers in V1 L1 at P21. Presynaptic VGlut1 (magenta) and postsynaptic marker PSD95 (green). Scale bar, 5 µm. ***B***, Density of colocalized puncta (left; *d* = 2.876, CI [1.392, 4.360]), VGlut1 puncta (middle; *d* = 1.136, CI [−0.348, 2.621]), and PSD95 puncta (right; *d* = 1.094, CI [−0.390, 2.578]) from V1 L1. ***C***, Representative ROIs labeled with VGlut1 and PSD95 in V1 L4 and (***D***) quantification of L4 colocalized (*d* = 1.326, CI [−0.158, 2.809]), VGlut1 (*d* = 0.129, CI [−1.355, 1.614]), and PSD95 (*d* = 1.060, CI [−0.424, 2.545]) puncta density. ***E***, Representative ROIs labeled with VGlut1 and PSD95 in V1 L5 and (***F***) quantification of L5 colocalized (*d* = 2.472, CI [0.988, 3.956]), VGlut1 (*d* = 1.474, CI [−0.010, 2.958]), and PSD95 (*d* = 1.461, CI [−0.023, 2.946]) puncta density. ***G***, Representative ROIs labeled with excitatory thalamocortical synapse markers in V1 L1 at P21. Presynaptic VGlut2 (magenta) and postsynaptic marker PSD95 (green). Scale bar, 5 µm. ***H***, Density of L1 colocalized puncta (left; *d* = 2.319, CI [0.835, 3.803]), VGlut2 puncta (middle; *d* = 2.081, CI [0.597, 3.565]), and PSD95 puncta (right; *d* = 0.841, CI [−0.643, 2.326]). ***I***, Representative ROIs labeled with VGlut2 and PSD95 in L4. ***J***, Density of L4 colocalized puncta (left; *d* = 1.606, CI [0.122, 3.090]), VGlut2 puncta (middle; *d* = 1.335, CI [−0.149, 2.819]), and PSD95 puncta (right; *d* = 0.037, CI [−1.447, 1.521]). For ***B***, ***D***, ***F***, ***H***, and ***J***: *n* = 6 sex-matched littermate pairs of control and cKO mice. In the control column, a triangle denotes WT mice and circle denotes cHet. Lines connect sex-matched control–cKO littermates. Dots represent per animal averages of 15 images. *p* values were calculated using a linear mixed-effects model. Effect size reported above as Cohen’s *d* with 95% confidence intervals (CI [lower, upper]). Refer to Extended Data Figure 6-1 for additional information on the analysis workflow. Refer to Extended Data Figure 6-2 for quantification of inhibitory synapse markers.

To determine whether astrocytic PTPRZ1 is required for proper excitatory synapse development, we quantified the density of colocalized excitatory synapse markers in different layers of V1 from sex-matched littermate pairs of *Ptprz1* cKO and control mice. We used different excitatory presynaptic markers in combination with excitatory postsynaptic marker PSD95 to differentiate between intracortical (VGlut1/PSD95) and thalamocortical (VGlut2/PSD95) synapses and collected high-resolution images via confocal microscopy. We used Synbot (Savage et al., 2024) with a stringent thresholding strategy (Extended Data Fig. 6-1) to quantify the number of synaptic puncta per image and defined synapses as the colocalization of pre- and postsynaptic markers. We performed analysis on V1 L1 and L4 of the visual cortex, where both excitatory input types are present, as well as L5, where the bulk of our morphology analysis was performed. *Ptprz1* cKO mice showed significant reductions in colocalization of excitatory intracortical synapse markers in L1 and L5 but not L4 (Fig. 6*A*–*F*). Colocalization of thalamocortical synapse markers was significantly reduced in L1 and L4 in *Ptprz1* cKO mice compared with controls (Fig. 6*G*–*J*). Analysis of presynaptic and postsynaptic puncta density suggested that, in some cases, the decrease in the density of colocalized synaptic markers might be driven by a decrease in presynaptic marker density. The density of VGlut1 puncta in L5 and VGlut2 puncta in L1 and L4 was significantly reduced in cKO mice compared with control, with no significant change in PSD95 density (Fig. 6*F*,*H*,*J*). We also examined the colocalization of inhibitory presynaptic marker VGAT and inhibitory postsynaptic marker gephyrin in L1, L4, and L5. In contrast to excitatory synapses, we did not observe any significant difference in colocalization of inhibitory synapse markers across layers (Extended Data Fig. 6-2*A*–*F*). However, we did observe an increase in colocalized inhibitory synapse markers in L5 that nearly reached statistical significance (*p* = 0.0501; Extended Data Fig. 6-2*F*). Collectively, these results demonstrate that loss of astrocytic *Ptprz1* leads to layer-specific reductions in the density of colocalized excitatory synapse markers in the V1 at P21.

10.1523/ENEURO.0386-25.2026.f6-1 Figure 6-1 Demonstration of thresholding strategy for synaptic marker analysis using Synbot. **A)** Left: Representative images of VGlut1 (top left, magenta) and PSD95 (bottom left, green) from P21 L1 visual cortex. Scale bar 10 µm. Right: binary images of each channel following thresholding. **B)** Top: Binary output of co-localized VGlut1 and PSD95 signal. Bottom: Merged output image generated by Synbot that contains merged channels (VGlut1 in magenta, PSD95 in green) and labeling of co-localized puncta (white dots). To demonstrate stringency of this analysis method, yellow arrows have been added to highlight examples of magenta and green puncta that are very close to each other, but do not overlap and are not counted by Synbot. Download Figure 6-1, TIF file.

10.1523/ENEURO.0386-25.2026.f6-2 Figure 6-2 Density of co-localized inhibitory synapse markers is unchanged at P21. **A)** Representative 17.5 µm x 17.5 µm regions of interest (ROIs) labeled with inhibitory synapse markers in V1 L1 at P21. Presynaptic VGAT (red) and postsynaptic marker gephyrin (cyan). Scale bar 5 µm. **B)** Density of co-localized puncta (left) (*d* = 0.527, CI [-0.957, 2.011]), VGAT puncta (middle) (*d* = 0.769, CI [-0.715, 2.253]), and Gephyrin puncta (right) (*d* = 0.214, CI [-1.270, 1.698]) from L1. **C)** Representative ROIs labeled with VGAT and gephyrin in L4 and **D)** quantification of co-localized (*d* = -0.444, CI [-1.928, 1.040]), VGAT (*d* = 1.091, CI [-0.393, 2.575]), and gephyrin (*d* = -0.415, CI [-1.899, 1.069]) puncta density. **E)** Representative ROIs labeled with VGAT and gephyrin in L5 and **F)** quantification of co-localized (*d* = -1.434, CI [-2.918, 0.049]), VGAT (*d* = 0.786, CI [-0.698, 2.270]), and gephyrin (*d* = -1.356, CI [-2.839, 0.128]) puncta density. For B, D, and F: n = 6 sex-matched littermate pairs of control and cKO mice. In the control column, a triangle denotes WT mice and circle denotes cHet. Lines connect sex-matched control-cKO littermates. Dots represent per animal averages of 15 images. P-values were calculated using a linear mixed effects model. Effect size reported above as Cohen’s d (*d*) with 95% Confidence Intervals (CI [lower, upper]). Download Figure 6-2, TIF file.

## Discussion

Here, we developed a new *Ptprz1* cKO mouse to study the astrocyte-specific functions of PTPRZ1 during brain development. We demonstrate successful astrocyte-specific postnatal deletion of all three PTPRZ1 isoforms and uncover the potential role of astrocytic PTPRZ1 in astrocyte morphogenesis and excitatory synapse development.

In our initial experiments with cortical astrocytes cocultured with cortical neurons, we observed a dramatic reduction in astrocyte branching complexity upon *Ptprz1* knockdown. However, the in vivo differences between *Ptprz1* cKO and corresponding control astrocytes were muted in comparison. At P21 we observed changes in astrocyte 3D architecture, with *Ptprz1* cKO astrocytes having altered ellipticity compared with control astrocytes, but no changes in astrocyte size or complexity. Several factors could explain this discrepancy between in vitro and in vivo findings: (1) Given the responsiveness of astrocytes to their microenvironment (Endo et al., 2022), genetic manipulations to astrocytes cocultured with neurons in 2D may produce different phenotypes than genetic manipulations to astrocytes in the 3D environment of the cortex, as other studies have shown (Baldwin et al., 2021; Rodriguez Salazar et al., 2025). (2) Sparse in vitro knockdown of *Ptprz1* allows for imaging of individually labeled astrocytes. In this scenario, knockdown astrocytes are surrounded by WT astrocytes that express PTPRZ1 and may compete with knockdown astrocytes for resources or space. In contrast, Cre-mediated deletion of *Ptprz1* from astrocytes in the developing cortex levels the playing field, enabling equal competition for space and resources. (3) Lastly, finer astrocyte leaflets are not resolvable by confocal microscopy (Baldwin et al., 2023). If PTPRZ1 regulates development of astrocyte leaflets and perisynaptic processes at the nanoscale, then super-resolution microscopy and/or electron microscopy would be required to visualize this phenotype.

Whether the changes we observed in astrocyte ellipticity are relevant to astrocyte function is unclear. Few studies have examined astrocyte ellipticity as a morphological metric, though a recent study noted changes in astrocyte ellipticity that coincided with transcriptional changes following traumatic brain injury (Gudenschwager-Basso et al., 2023). Visually, the decrease in prolate ellipticity appears to be driven by the decreased propensity of *Ptprz1* cKO astrocytes to extend their branches outside of spherical arbor (as shown in the representative images in Fig. 4*B*). This could be reflective of abnormal engagement of *Ptprz1* cKO astrocytes with their tissue microenvironment. Future studies are necessary to determine whether alterations to astrocyte ellipticity reflect changes in molecular and/or functional states.

Elucidating the role of astrocytic PTPRZ1 in excitatory synapse formation also requires further investigation. In the present study, we performed a rigorous analysis of the density of colocalized pre- and postsynaptic synapse markers for three different synapse types in V1 at P21. We found decreases in the density of colocalized markers labeling excitatory intracortical synapses in L1 and L5 and excitatory thalamocortical synapses in L1 and L4. We applied a stringent thresholding strategy and used Synbot to identify overlapping pre- and postsynaptic puncta. This strategy has been thoroughly vetted (Savage et al., 2024) and has previously been shown to reflect functional synapse deficits via slice electrophysiology and structural synapse deficits via decreased dendritic spine number using both electron and confocal microscopy (Risher et al., 2018). To provide additional rigor to this analysis, we analyzed synaptic data using a linear mixed-effects model to account for any variability of developmental conditions (i.e., litter; Jimenez and Zylka, 2021) on the synapse number, as well as technical variation between sample preparation and imaging sessions. Our findings therefore provide strong evidence of a decrease in excitatory synapse density following astrocyte-specific *Ptprz1* deletion. To corroborate these findings, additional future experiments, such as analysis of dendritic spine density or functional characterization by electrophysiology, will be useful.

Uncovering the mechanistic function of astrocytic PTPRZ1 at excitatory synapses is another important avenue for further investigation. Although defects in astrocyte morphogenesis are associated with impaired synapse formation and/or function (Stogsdill et al., 2017; Cheng et al., 2023), an absence of morphological phenotype does not preclude synaptic deficits. In the case of PTPRZ1, the minor morphological differences that we observed in *Ptprz1 *cKO mice at the level of confocal microscopy do not suggest an obvious link to excitatory synapse development. Because we observed PTPRZ1 protein localization at excitatory synapses in WT mice, we reason that, in the absence of significant changes to astrocyte morphology, the absence of PTPRZ1 protein itself at the synapse could be the cause of any synaptic defects. PTPRZ1 could act directly at neuronal synapses to promote synapse formation as a secreted factor (phosphacan) and/or as a transmembrane receptor. Several PTPRZ1 binding partners have been described, including pleiotrophin (PTN), a secreted growth factor that regulates various aspects of nervous system development (Gonzalez-Castillo et al., 2014). Whether any astrocyte-specific PTPRZ1 functions during brain development are PTN-dependent is an exciting topic for future investigation, particularly given the interest in PTN as a therapeutic target to treat nervous system injury (Pushpam et al., 2024; Lei et al., 2025), alcohol use disorder (Liran et al., 2020), and other neurological disorders (Pastor et al., 2018; Brandebura et al., 2025).

In addition to astrocytes, PTPRZ1 is expressed in various cell types during early nervous system development, including RGCs and OPCs (Zhang et al., 2014; Zhang et al., 2016; Loo et al., 2019). Several studies suggest important functions for PTPRZ1 in nervous system development and plasticity across multiple cell types, yet the cell-type–specific functions of PTPRZ1 are unknown. This mouse model will be a useful tool for cell-type–specific and temporal deletion of *Ptprz1* to understand its function in healthy brain development. Importantly, PTPRZ1 is emerging as a therapeutic target for a growing list of neurological and neuropsychiatric disorders, including schizophrenia, glioblastoma, and substance use disorder (Fernandez-Calle et al., 2018; Pastor et al., 2018; Nagai et al., 2022; Papadimitriou and Kanellopoulou, 2023). Thus, understanding the cell-type–specific functions of PTPRZ1 is essential to develop effective therapeutic strategies to target nervous system dysfunction while mitigating off-target consequences of manipulating PTPRZ1 function.

## Synthesis

Reviewing Editor: Katrina Choe, McMaster University Decisions are customarily a result of the Reviewing Editor and the peer reviewers coming together and discussing their recommendations until a consensus is reached. When revisions are invited, a fact-based synthesis statement explaining their decision and outlining what is needed to prepare a revision will be listed below. The following reviewer(s) agreed to reveal their identity: NONE.

In this manuscript the authors investigate the astrocyte-specific functions of PTPRZ1, one of the most abundant astrocytic proteins during development. They combine an in vitro knockdown approach with a conditional knockout mouse model, which required generation of a floxed Ptprz1 mouse and crossing to the astrocyte and radial glial-specific Aldh1l1-CreER line. They report that PTPRZ1 knockdown in cultured astrocytes markedly alters morphological complexity, whereas in vivo, only astrocyte ellipticity is affected. Cortical cell numbers and layering were unaffected. The authors also identify potential changes in synapse density following astrocytic Ptprz1 deletion.

Both reviewers agreed that this is a timely and important study and that experiments were thorough and largely rigorous. Reviewers also agreed that the generation of the new floxed Ptprz1 line is commendable and likely to be very useful for many future studies. However, both reviewers shared concerns about the approaches used to analyze synapse status in the context of astrocyte Ptprz1 loss. They also raised additional methodological concerns.

Major concerns:

1. Immunohistochemistry for synaptic proteins in vivo is a highly variable measurement. If the authors really want to make the point that excitatory synapse density is decreased in vivo, they should add mEPSCs measured from ex vivo slices to support this claim. Alternatively, they could virally express a fluorescent reporter in neurons to assess postsynaptic spine density. The alternative to performing these experiments would be to greatly tone down the conclusions and discuss the need for confirming these observations via electrophysiology, dendritic spine analysis, or electron microscopy approaches.

2. Along these same lines, the vGlut1 immunostaining in Figures 5B,C and Figure 6 is rather diffuse and does not show clearly defined puncta similar to those observed with VGlut2 signal. This raises concerns about the accuracy and validity of the quantification approach. The authors must demonstrate that their vGlut1 staining reliably labels discrete synapses and provide evidence that their quantification method is appropriate for this marker.

3. The use of paired t-tests for measurements taken from separate animals (Figures 3 and 6) is highly unusual. Paired t-test is typically only used for data points that came from the same animal (e.g. baseline behavior and behavior of the same animal following drug treatment; e.g. comparison of data from two different brain regions or two different cell types that came from the same animal). These data should be reanalyzed using an appropriate unpaired statistical approach.

4. To fully illustrate fidelity of their in vivo astrocyte PTPRZ1 KO, the authors should include immunostaining of Ptprz1 in other cell types (especially neurons and OPCs), or qPCR based analysis from purified cell types, to show that depletion is limited to astrocytes only. PMID: 30096299, PMID: 25186741, and PMID: 26523646 all indicate that some expression in oligodendrocyte lineage cells is to be expected.

5. Although the authors report no difference in the final number of astrocytes, it remains unclear whether the timing of astrocyte proliferation is altered. A delayed proliferative wave could yield similar endpoint numbers while still reflecting a biologically meaningful deficit. The study lacks a developmental time course (e.g., P7, P14, or earlier) and does not include BrdU/EdU incorporation assays to quantify proliferation at defined stages. Without these data, it is difficult to conclude that loss of PTPRZ1 does not impact astrogliogenesis. A proper temporal assessment of proliferation in cKO versus WT animals is necessary to support this claim.

6. The authors justify focusing on layer V of the visual cortex by stating that PTPRZ1 expression is higher there, yet the manuscript does not provide staining data supporting this claim. Moreover, in Figures 4J and 4K, the authors also analyze layers I and V, noting that PTPRZ1 is highly expressed in layer I as well. If both layers exhibit high expression, the morphological analyses (volume, sphericity, ellipticity, and related measures) should be performed in layer I in addition to layer V. Similarly, for Figure 5, the rationale for selecting the specific brain regions analyzed is unclear- are these also within the visual cortex? Clear justification and supporting expression data are needed to validate the choice of cortical layers and regions included in the analysis.

Minor concerns:

- Authors may want to include a graph (adapted from a public dataset is fine) showing relative levels of Ptprz1 expression in different brain cell types.

- The manuscript does not report the number of males and females used in each experiment. This information should be clearly provided for all datasets.

- The in vitro experiments rely on rat astrocytes cultured in the presence of fetal bovine serum (FBS), a condition that does not recapitulate the extracellular environment in which astrocytes normally proliferate or mature in vivo. Serum exposure is well known to induce a reactive, de-differentiated state in astrocytes and can significantly alter their morphology, gene expression, and signaling properties. Additionally, the isolation method used does not yield highly pure astrocyte populations (compared to immunopanning, MACS/FACS-purified astrocytes). The authors should more clearly acknowledge these limitations discussing conclusions drawn from these experiments.

- Statistical reporting is incomplete: only p-values are listed. Please include descriptive statistics (mean, SEM/SD, n, and effect sizes when appropriate).

- The source of the HEK293 cells used in the study should be specified.

- Please indicate the FIJI plugin and version used for the Sholl analysis.

- The term "peak complexity" (line 221) requires a clear definition.

- Antibodies should be moved to the immunohistochemistry section rather than appearing under cell counting for clarity.

- RRIDs should be added for all antibodies, cell lines, and other reagents.

- The manuscript inconsistently reports antibody immunoglobulin types (e.g., IgG2, IgG). Please decide whether to include these details and apply the choice consistently throughout.

- Line 300 refers to "Imaris analysis," yet the methodological description in this section refers exclusively to ImageJ/FIJI. Please clarify which software and analysis pipeline were actually used.

- Line 398: The secondary antibody is anti-chicken, not anti-chicken-GFP. Please revise.

- For the Western blot analysis, clarify whether left or right hemispheres were used consistently and whether hemisphere selection was randomized.

- Line 480 (and line 672): Once astrocytes have been passaged, they are no longer considered primary. Please remove the term "primary" and specify that these are cortical astrocytes/neurons.

- A suggestion rather than a critique: The authors highlight the importance of analyzing and interpreting the data using the animal as an experimental unit instead of a cell, a practice that is unfortunately common in the field and we should abandon as a community. For clarity, they included the analysis per cell in the extended data, which is appreciated. The authors are encouraged to use superplots in future publications (Lord et al 2020).

- In Figures 5B-E, the red and magenta channels are difficult to distinguish. A more clearly separable color scheme would improve visualization.

## Author Response

Response to Reviewers We are grateful to the reviewers for their constructive feedback and recommendations for improving the manuscript. We are delighted that both reviewers found that the experiments presented in the manuscript are timely, important to the field, and largely rigorous while providing an important new resource in the Ptprz1 conditional knockout mouse. Major concerns centered on conclusions drawn from immunohistochemistry-based synapse density analysis, choice of statistical tests used in some experiments, lack of confirmation of PTPRZ1 expression in non-astrocyte cell types known to express PTPRZ1, conclusions regarding astrogliogenesis drawn from cell counting experiments, and insufficient justification for layer-specific analyses. In response, we have revised the manuscript by: 1) performing additional analyses demonstrating the fidelity of the synaptic puncta counting strategy revising our language surrounding the conclusion; 2) changing our approach for statistical analysis of sex-matched littermate pairs, 3) adding additional validation of astrocyte-specific Ptprz1 deletion during development by demonstrating unaffected PTPRZ1 expression in OPCs and neurons; and 4) revising the text to address concerns regarding astrogliogenesis, layer-specific analysis, and other minor points. Below we provide a point-by-point response to each reviewer comment. Changes to the manuscript text are visible in the "tracked changes" version of the revised manuscript file.

Major Concerns Immunohistochemistry for synaptic proteins in vivo is a highly variable measurement. If the authors really want to make the point that excitatory synapse density is decreased in vivo, they should add mEPSCs measured from ex vivo slices to support this claim. Alternatively, they could virally express a fluorescent reporter in neurons to assess postsynaptic spine density. The alternative to performing these experiments would be to greatly tone down the conclusions and discuss the need for confirming these observations via electrophysiology, dendritic spine analysis, or electron microscopy approaches.

We have revised the manuscript text to significantly tone down the conclusions surrounding excitatory synapse density. In the discussion section, we discuss the need for additional studies to support these observations.

Along these same lines, the vGlut1 immunostaining in Figures 5B,C and Figure 6 is rather diffuse and does not show clearly defined puncta similar to those observed with VGlut2 signal. This raises concerns about the accuracy and validity of the quantification approach. The authors must demonstrate that their vGlut1 staining reliably labels discrete synapses and provide evidence that their quantification method is appropriate for this marker.

We appreciate the reviewers bringing this to our attention as it caused us to revisit and critically review all of the raw data files containing VGlut1 immunolabeling and improve the quality of our analysis. While it is typical that a large percentage of presynaptic signal will not co-localize with PSD95 (McKinstry et al., 2014; Risher et al., 2018; Wilton et al., 2023; Barron et al., 2024; Szewczyk et al., 2024; Brandebura et al., 2025), we agree that the representative images that we used are of insufficient quality for the reader to critically evaluate the data.

Figure 5:

We reexamined our raw STED data files and confirmed our deconvolution settings. We noticed that the signal-to-noise ratio was lower for VGlut1 and PTPRZ1 channels compared to VGlut2. STED channels that have lower signal-to-noise ratio require additional adjustments to brightness and contrast to improve visualization of real signal and we failed to account for this during our initial figure preparation. We therefore adjusted these channels to reduce background noise.

Figure 6:

Upon careful review of the original data and analysis files, we realized that the VGlut1/PSD95 data set had not been analyzed with Synbot and had instead been analyzed with an older puncta analyzer plugin (Synbot was not yet published at the time of analysis). In contrast, the VGlut2/PSD95 and VGAT/Gephyrin data sets were analyzed with Synbot, which includes a Noise Reduction step prior to thresholding. For consistency and improved data quality, we reanalyzed all of the VGlut1/PSD95 data using Synbot and included the noise reduction step (Gaussian blur = 0.570, Subtract background rolling ball radius = 50). We also determined that the VGlut1 staining in one of the pairs was of poor quality and not suitable for analysis. Because we did not have any additional visual cortex sections for this pair, it was excluded from analysis. We generated a new sex-matched littermate pair to replace this sample for the VGlut1/PSD95 data and included this pair in our analysis presented in Figure 6.

To demonstrate the fidelity of this analysis method and demonstrate the stringency of our threshold setting, we also include a new Extended Data Figure 6-1 that shows examples of binary thresholded images for VGlut1 and PSD95 as well as examples of output files that mark co-localized puncta. We note that additional examples of analysis using Synbot, as well as a comparison between Synbot and other methods of quantifying synaptic markers are available in a published study (Savage et al., 2024).

The use of paired t-tests for measurements taken from separate animals (Figures 3 and 6) is highly unusual. Paired t-test is typically only used for data points that came from the same animal (e.g. baseline behavior and behavior of the same animal following drug treatment; e.g. comparison of data from two different brain regions or two different cell types that came from the same animal). These data should be reanalyzed using an appropriate unpaired statistical approach.

We thank the reviewers for raising this as a point of discussion. We originally used the paired t-test as a way to control for the random effects of litter. Previous studies have discussed the need to consider litter effects (from litter size, maternal rearing behavior, etc.) particularly in neurodevelopmental studies (Lazic and Essioux, 2013; Jimenez and Zylka, 2021). In our experiment, a paired t-test treats each pair as an experimental unit and measures the magnitude of difference between the two. It is statistically equivalent to an unpaired t-test of a dataset where the control has been set to 100 for each pair, and the experimental group is presented as a percentage of the control, a strategy that has been used by others to analyze this type of data (Blanco-Suarez et al., 2018; Brandebura et al., 2025). Though we believe that the paired t-test is a valid statistical approach for controlling for random effects when experiments are performed with discreet pairs, we could not find a clear reference in the literature to support this idea. We instead chose to use a linear mixed model as the literature supports the use of a linear mixed effects model as a valid statistical approach for controlling for litter effects (Jimenez and Zylka, 2021). We reanalyzed all of our paired datasets (synapse and cell count) in R Studio using a linear mixed model. We provide additional details in the methods section and include the R script on our Github site. For the graphs in the figures, we have kept the lines connecting the sex-matched littermate pairs to provide additional transparency.

As a point of comparison, we include below a table showing the p-values obtained for each dataset from the linear mixed effects model, a paired t-test, and an unpaired t-test. In all but two instances (bolded in the table below), the statistical approach used did not change the overall conclusions drawn from the data. In some instances, the p-value is higher with the unpaired t-test while in others it is lower. Note that the VGlut1 values used below come from the reanalyzed dataset discussed above.

While it would be simpler for us to say that we will switch everything to an unpaired t-test, we believe that developmental datasets should consider random effects that may impact data. We therefore favor the linear mixed model. Indeed, as Jimenez and Zylka found, litter effect was stronger than treatment in their experiments, and masked key phenotypes. While this is not the case for our data, as we found no effect of litter, we still advocate for use of this model as a valid statistical approach and hope that our study can serve as a reference for others to improve statistical rigor in developmental studies.

Table: p-values obtained from different statistical tests To fully illustrate fidelity of their in vivo astrocyte PTPRZ1 KO, the authors should include immunostaining of Ptprz1 in other cell types (especially neurons and OPCs), or qPCR based analysis from purified cell types, to show that depletion is limited to astrocytes only. PMID: 30096299, PMID: 25186741, and PMID: 26523646 all indicate that some expression in oligodendrocyte lineage cells is to be expected.

To conditionally delete Ptprz1 from astrocytes in the developing cortex, we crossed our Ptprz1 floxed mice the Aldh1l1CreERT2 transgenic mouse line and used a previously published tamoxifen administration strategy that has been extensively validated (Baldwin et al., 2021). According to this prior study "This protocol efficiently and specifically targeted astrocytes in the mouse cortex with 95.6% {plus minus} 0.8% (mean {plus minus} SEM) of astrocytes (16,511 cells counted from 15 animals) and only 0.56% {plus minus} 0.06% of neurons (92,555 cells counted from 15 animals) expressing tdTomato." To provide additional evidence that our Ptprz1 deletion is specific to astrocytes, we performed immunolabeling for PTPRZ1 and PDGFRα (to label OPCs) or β3-Tubulin (to label neurons) in Ptprz1 WT and cKO mice in P21-22 primary visual cortex. According to previous transcriptomic studies that we reference in Extended Data Figure 1-1A-B (Zhang et al., 2014; Zhang et al., 2016), OPCs express the highest level of Ptprz1 after astrocytes. There is also a low level of expression of Ptprz1 in neurons. We observed consistent PTPRZ1 expression in PDGFRα-expressing cells in both WT and cKO mice. In our new extended data figure 2-1 we show representative images of L5 visual cortex (Extended Data Figure 2-1A) and corpus callosum (2-1B). Consistent with transcriptomic studies, we observed minimal PTPRZ1 protein expression in β3-Tubulin+ neurons in both WT and cKO mice. Of note, neuronal expression of PTPRZ1 appeared highest in axons in both WT and cKO mice, as evidence by the images of corpus callosum presented in Extended Data Figure 2-1D.

Although the authors report no difference in the final number of astrocytes, it remains unclear whether the timing of astrocyte proliferation is altered. A delayed proliferative wave could yield similar endpoint numbers while still reflecting a biologically meaningful deficit. The study lacks a developmental time course (e.g., P7, P14, or earlier) and does not include BrdU/EdU incorporation assays to quantify proliferation at defined stages. Without these data, it is difficult to conclude that loss of PTPRZ1 does not impact astrogliogenesis. A proper temporal assessment of proliferation in cKO versus WT animals is necessary to support this claim.

We thank the reviewers for raising this concern so that we can avoid over interpretation of our data. The reviewers are correct that our data only supports a claim of no change in final astrocyte numbers at P21. Because we did not investigate astrocyte proliferation or astrogliogenesis in this study, we have significantly revised the text to clarify what we were measuring with our experiment (total number of astrocytes at P21) and the conclusion we can make from our results (no difference). To provide additional context to readers, we also include information in the text on the percentage of dividing glial cells in the mouse cortex at different developmental timepoints from a previous study (Ge et al., 2012). Based on the timing of our Ptprz1 deletion, we would expect that between 10 -15% or astrocytes are still in a proliferative state by the time the protein is gone.

The authors justify focusing on layer V of the visual cortex by stating that PTPRZ1 expression is higher there, yet the manuscript does not provide staining data supporting this claim. Moreover, in Figures 4J and 4K, the authors also analyze layers I and V, noting that PTPRZ1 is highly expressed in layer I as well. If both layers exhibit high expression, the morphological analyses (volume, sphericity, ellipticity, and related measures) should be performed in layer I in addition to layer V. Similarly, for Figure 5, the rationale for selecting the specific brain regions analyzed is unclear- are these also within the visual cortex? Clear justification and supporting expression data are needed to validate the choice of cortical layers and regions included in the analysis.

We have edited the text to explain why specific layers were chosen for each experiment. We clarify that PTPRZ1 appears robustly expressed in all layers of the visual cortex at P21 and even at P7 (Figure 2, Extended Data Figure 2-2). We target V1 L5 for detailed morphology analyses because our viral labeling strategy is most efficient in targeting deeper layer astrocytes and there is a large body of literature investigating astrocyte morphology in this layer. In our overview images of PTPRZ1 expression at P21 (Figure 2C), we noticed that PTPRZ1 expression seemed particularly strong in L1. Because we had already performed analysis of synaptic marker density in L1, reviewers from a previous journal asked us to perform morphology analysis in L1. We also found it interesting to examine L1 astrocyte morphology as L1 astrocytes are molecularly distinct from other cortical layers (Bocchi et al., 2025). Unfortunately, our viral labeling strategy is not very effective labeling L1 astrocytes. Due to this technical limitation, we exhausted our tissue sections in a search for L1 astrocytes that were suitable for data collection. While we were able to collect enough cells for analysis of neuropil infiltration volume (NIV) (which can be performed as long as ~20 µm of the astrocyte is present within the section in the z dimension), we were not able to collect enough complete astrocytes for 3D morphology analysis. We have added text to the results section to describe this limitation. Since we found no impact of Ptprz1 cKO on NIV (Extended Data Figure 4-1, L-M) and only mild impacts of Ptprz1 deletion to V1 L5 astrocyte morphology, we predict minimal impact to V1 L1 astrocyte morphology yet acknowledge this to be an outstanding question.

For Figure 5, we have provided additional in-text justification for layer-specific data collection in the visual cortex. We state that, "We focused on V1 L1 and L5 excitatory intracortical synapses due to the strong expression of PTPRZ1 in L1, the bulk of our morphology analyses being conducted in L5, and the abundance of this synapse type in these layers. We examined excitatory thalamocortical synapses in V1 L1 and L4 due to the preferential targeting of excitatory thalamocortical inputs specifically to these layers." The layers chosen for Figure 5 are also consistent with the layers that we chose for synapse analysis in Figure 6, for the same reasons.

Minor Concerns - Authors may want to include a graph (adapted from a public dataset is fine) showing relative levels of Ptprz1 expression in different brain cell types." A graph comparing Ptprz1 expression in different brain cell types in mouse and human is presented in Extended Data Figure 1-1. Panel A shows mouse data adapted from Zhang et al., 2014. Panel B shows human data adapted from Zhang et al., 2016.

- The manuscript does not report the number of males and females used in each experiment. This information should be clearly provided for all datasets.

We have added the number of males and females to the detailed methods section for each experiment yielding quantitative datasets.

- "The in vitro experiments rely on rat astrocytes cultured in the presence of fetal bovine serum (FBS), a condition that does not recapitulate the extracellular environment in which astrocytes normally proliferate or mature in vivo. Serum exposure is well known to induce a reactive, de-differentiated state in astrocytes and can significantly alter their morphology, gene expression, and signaling properties. Additionally, the isolation method used does not yield highly pure astrocyte populations (compared to immunopanning, MACS/FACS-purified astrocytes). The authors should more clearly acknowledge these limitations discussing conclusions drawn from these experiments." We fully agree that astrocytes cultured on their own and/or in serum are not representative of astrocytes in vivo and would be more comparable to reactive astrocytes. Therefore, all of our morphological analysis is performed on astrocytes co-cultured with neurons in serum-free conditions. We elaborate here on the methods to provide additional clarity on the procedure.

Prior to co-culture, astrocytes and neurons are isolated from the same litter of P1 rats and cultured independently. Neurons are isolated by immunopanning and cultured in serum-free conditions supplemented with growth factors. The purity of these neuron cultures has been demonstrated in several previous studies(Stogsdill et al., 2017; Risher et al., 2018; Irala et al., 2024). Astrocytes are isolated by a combination of shaking and fibroblast elimination and cultured in serum in accordance with previous protocols that have also demonstrated the purity of this approach (McCarthy and de Vellis, 1980; Stogsdill et al., 2017). Astrocytes cultured on their own and in serum using this method display a fibroblast-like morphology with few branches, though they express GFAP and other astrocyte markers such as GLT1, glutamine synthetase, and Connexin 43 (Stogsdill et al., 2017; Baldwin et al., 2021).

However, following trypsinization and co-culture upon neurons for 48-72 hours in serum-free neuronal media, astrocytes develop a complex, branched morphology. This enhanced morphology provides ample resolution for screening factors that may impact astrocyte morphogenesis. This approach has been used in several published studies to uncover potential regulators of astrocyte morphogenesis for further in vivo study and for screening of molecular pathways regulating astrocyte morphogenesis. (Stogsdill et al., 2017; Baldwin et al., 2021; Tan et al., 2023; Faust et al., 2025; Rodriguez Salazar et al., 2025).

- Statistical reporting is incomplete: only p-values are listed. Please include descriptive statistics (mean, SEM/SD, n, and effect sizes when appropriate).

We have added descriptive statistics to the figure legends where appropriate.

- The source of the HEK293 cells used in the study should be specified.

Added this information to the methods.

- Please indicate the FIJI plugin and version used for the Sholl analysis.

Added this information to the methods.

- The term "peak complexity" (line 221) requires a clear definition.

Revised the methods to define peak complexity.

- Antibodies should be moved to the immunohistochemistry section rather than appearing under cell counting for clarity.

Given the large number of antibodies that are used in this study, we included the general procedure for immunohistochemistry and then specify the primary antibodies in each subsection (cell counting, Astrocyte 3D morphology, etc.). In this first section, we write that the specifics on primary antibodies are detailed in each subsection. We find this arrangement helpful in directing readers to the specific use case for each antibody and in minimizing ambiguity if different concentrations of antibody were used for different approaches.

- RRIDs should be added for all antibodies, cell lines, and other reagents.

We have added RRIDs for all antibodies, cell lines, and other reagents for which RRIDs are available.

- The manuscript inconsistently reports antibody immunoglobulin types (e.g., IgG2, IgG). Please decide whether to include these details and apply the choice consistently throughout.

We have added the immunoglobulin isotypes to secondary antibodies throughout. For mouse monoclonal primary and goat anti-mouse secondary antibodies, we also include the isotype subgroup (e.g., IgG1, IgG2a) as we use goat anti-mouse secondary antibodies that detect only the specific isotype subgroup and are cross adsorbed against the others. The use of isotype subgroup-specific secondaries greatly diminishes mouse-on-mouse background staining.

Of note, the isotype subgroup cannot be specified for polyclonal primary antibodies, as these antibodies by nature contain several subgroups. Additionally, secondary antibodies that detect specific isotype subgroups are unavailable for species other than mouse. Thus, isotype subgroup information is included only for mouse IgG antibodies.

- Line 300 refers to "Imaris analysis," yet the methodological description in this section refers exclusively to ImageJ/FIJI. Please clarify which software and analysis pipeline were actually used.

Thank you for spotting this error. We removed the incorrect reference to Imaris. The analysis of cell number was performed in Cell Profiler, as specified earlier in this subsection.

- Line 398: The secondary antibody is anti-chicken, not anti-chicken-GFP. Please revise.

Revised.

- For the Western blot analysis, clarify whether left or right hemispheres were used consistently and whether hemisphere selection was randomized.

Hemisphere selection was randomized. We have updated the methods accordingly.

- Line 480 (and line 672): Once astrocytes have been passaged, they are no longer considered primary. Please remove the term "primary" and specify that these are cortical astrocytes/neurons.

We have removed "primary" and specified that these are cortical astrocytes and cortical neurons.

- A suggestion rather than a critique: The authors highlight the importance of analyzing and interpreting the data using the animal as an experimental unit instead of a cell, a practice that is unfortunately common in the field and we should abandon as a community. For clarity, they included the analysis per cell in the extended data, which is appreciated. The authors are encouraged to use superplots in future publications (Lord et al 2020).

We appreciate this suggestion. We will consider this format for our future studies.

- In Figures 5B-E, the red and magenta channels are difficult to distinguish. A more clearly separable color scheme would improve visualization.

Thank you for this suggestion. We have revised the color scheme for improved visualization of discrete channels.

References Baldwin KT, Tan CX, Strader ST, Jiang C, Savage JT, Elorza-Vidal X, Contreras X, Rulicke T, Hippenmeyer S, Estevez R, Ji RR, Eroglu C (2021) HepaCAM controls astrocyte self-organization and coupling. Neuron 109:2427-2442 e2410.

Barron JJ, Mroz NM, Taloma SE, Dahlgren MW, Ortiz-Carpena JF, Keefe MG, Escoubas CC, Dorman LC, Vainchtein ID, Chiaranunt P, Kotas ME, Nowakowski TJ, Bender KJ, Molofsky AB, Molofsky AV (2024) Group 2 innate lymphoid cells promote inhibitory synapse development and social behavior. Science 386:eadi1025.

Blanco-Suarez E, Liu TF, Kopelevich A, Allen NJ (2018) Astrocyte-Secreted Chordin-like 1 Drives Synapse Maturation and Limits Plasticity by Increasing Synaptic GluA2 AMPA Receptors. Neuron.

Bocchi R, Thorwirth M, Simon-Ebert T, Koupourtidou C, Clavreul S, Kolf K, Della Vecchia P, Bottes S, Jessberger S, Zhou J, Wani G, Pilz GA, Ninkovic J, Buffo A, Sirko S, Gotz M, Fischer-Sternjak J (2025) Astrocyte heterogeneity reveals region-specific astrogenesis in the white matter. Nat Neurosci 28:457-469.

Brandebura AN, Paumier A, Asbell QN, Tao T, Micael MKB, Sanchez S, Allen NJ (2025) Dysregulation of astrocyte-secreted pleiotrophin contributes to neuronal structural and functional deficits in Down syndrome. Cell Rep 44:116300.

Faust TE, Lee YH, O'Connor CD, Boyle MA, Gunner G, Duran-Laforet V, Ferrari LL, Murphy RE, Badimon A, Sakers K, Eroglu C, Ayata P, Schaefer A, Schafer DP (2025) Microglia-astrocyte crosstalk regulates synapse remodeling via Wnt signaling. Cell 188:5212-5230 e5221.

Ge WP, Miyawaki A, Gage FH, Jan YN, Jan LY (2012) Local generation of glia is a major astrocyte source in postnatal cortex. Nature 484:376-380.

Irala D, Wang S, Sakers K, Nagendren L, Ulloa Severino FP, Bindu DS, Savage JT, Eroglu C (2024) Astrocyte-secreted neurocan controls inhibitory synapse formation and function. Neuron 112:1657-1675 e1610.

Jimenez JA, Zylka MJ (2021) Controlling litter effects to enhance rigor and reproducibility with rodent models of neurodevelopmental disorders. J Neurodev Disord 13:2.

Lazic SE, Essioux L (2013) Improving basic and translational science by accounting for litter-to-litter variation in animal models. BMC Neurosci 14:37.

McCarthy KD, de Vellis J (1980) Preparation of separate astroglial and oligodendroglial cell cultures from rat cerebral tissue. J Cell Biol 85:890-902.

McKinstry SU, Karadeniz YB, Worthington AK, Hayrapetyan VY, Ozlu MI, Serafin-Molina K, Risher WC, Ustunkaya T, Dragatsis I, Zeitlin S, Yin HH, Eroglu C (2014) Huntingtin is required for normal excitatory synapse development in cortical and striatal circuits. J Neurosci 34:9455-9472.

Risher WC, Kim N, Koh S, Choi JE, Mitev P, Spence EF, Pilaz LJ, Wang D, Feng G, Silver DL, Soderling SH, Yin HH, Eroglu C (2018) Thrombospondin receptor alpha2delta-1 promotes synaptogenesis and spinogenesis via postsynaptic Rac1. J Cell Biol 217:3747-3765.

Rodriguez Salazar MP, Kolanukuduru S, Ramirez V, Lyu B, Manigault G, Sejourne G, Sesaki H, Yu G, Eroglu C (2025) Mitochondrial fission controls astrocyte morphogenesis and organization in the cortex. J Cell Biol 224.

Savage JT, Ramirez JJ, Risher WC, Wang Y, Irala D, Eroglu C (2024) SynBot is an open-source image analysis software for automated quantification of synapses. Cell Rep Methods 4:100861.

Stogsdill JA, Ramirez J, Liu D, Kim YH, Baldwin KT, Enustun E, Ejikeme T, Ji RR, Eroglu C (2017) Astrocytic neuroligins control astrocyte morphogenesis and synaptogenesis. Nature 551:192-197.

Szewczyk LM, Lipiec MA, Liszewska E, Meyza K, Urban-Ciecko J, Kondrakiewicz L, Goncerzewicz A, Rafalko K, Krawczyk TG, Bogaj K, Vainchtein ID, Nakao-Inoue H, Puscian A, Knapska E, Sanders SJ, Jan Nowakowski T, Molofsky AV, Wisniewska MB (2024) Astrocytic beta-catenin signaling via TCF7L2 regulates synapse development and social behavior. Mol Psychiatry 29:57-73.

Tan CX, Bindu DS, Hardin EJ, Sakers K, Baumert R, Ramirez JJ, Savage JT, Eroglu C (2023) delta-Catenin controls astrocyte morphogenesis via layer-specific astrocyte-neuron cadherin interactions. J Cell Biol 222.

Wilton DK, Mastro K, Heller MD, Gergits FW, Willing CR, Fahey JB, Frouin A, Daggett A, Gu X, Kim YA, Faull RLM, Jayadev S, Yednock T, Yang XW, Stevens B (2023) Microglia and complement mediate early corticostriatal synapse loss and cognitive dysfunction in Huntington's disease. Nat Med 29:2866-2884.

Zhang Y, Chen K, Sloan SA, Bennett ML, Scholze AR, O'Keeffe S, Phatnani HP, Guarnieri P, Caneda C, Ruderisch N, Deng S, Liddelow SA, Zhang C, Daneman R, Maniatis T, Barres BA, Wu JQ (2014) An RNA-sequencing transcriptome and splicing database of glia, neurons, and vascular cells of the cerebral cortex. J Neurosci 34:11929-11947.

Zhang Y, Sloan SA, Clarke LE, Caneda C, Plaza CA, Blumenthal PD, Vogel H, Steinberg GK, Edwards MS, Li G, Duncan JA, 3rd, Cheshier SH, Shuer LM, Chang EF, Grant GA, Gephart MG, Barres BA (2016) Purification and Characterization of Progenitor and Mature Human Astrocytes Reveals Transcriptional and Functional Differences with Mouse. Neuron 89:37-53.
