# Supplementary figures and images for: Astrocyte-Derived PTPRZ1 Regulates Excitatory Synapse Density in the Mouse Cortex
Source: eNeuro. 2026 Apr 21;13(4):ENEURO.0386-25.2026. doi: 10.1523/ENEURO.0386-25.2026 (PMC13102477; doi:10.1523/ENEURO.0386-25.2026)

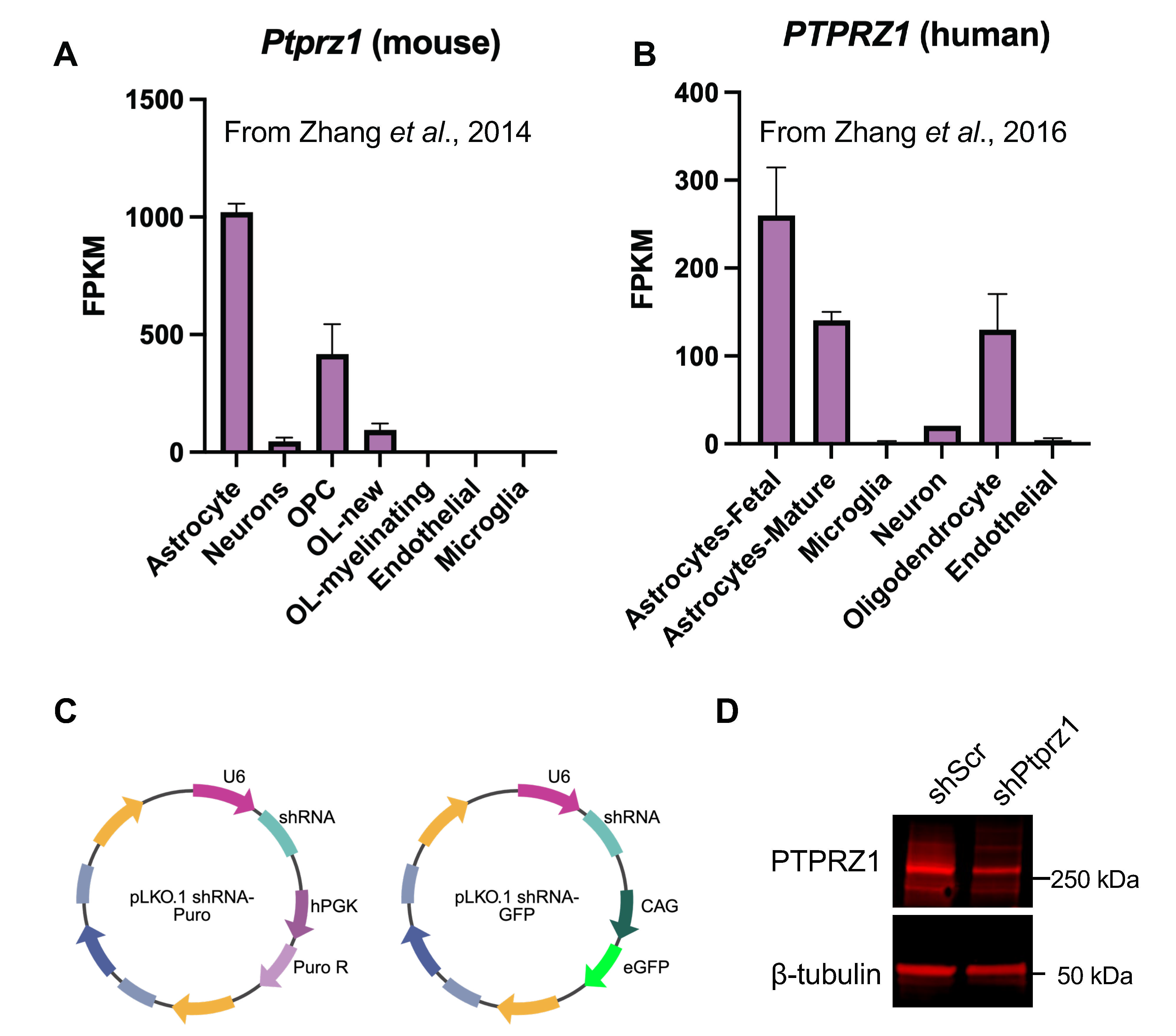

Supplement: Figure 1-1 — Ptprz1 expression and shRNA validation. A) Ptprz1 gene expression levels per cell type in P7 mouse cortex from Zhang et al., 2014. B) PTPRZ1 gene expression levels per cell type in human from Zhang et al., 2016. C) Plasmid maps for pLKO.1 vectors used in this study. Plasmids expressing shRNA and puromycin resistance (PuroR) were packaged into lentivirus and transduced into astrocytes to validate shRNA knockdown efficiency (pLKO.1 shRNA-Puro). For morphology analysis, the hPGK promoter and Puro R were replaced with a CAG promoter driving expression of eGFP (pLKO.1 shRNA-GFP). Maps created with BioRender.com. D) Western blot of primary rat astrocytes transduced with lentivirus expressing pLKO.1 shScr-Puro or pLKO.1 shPtprz1-Puro and treated with puromycin to eliminate non-transduced astrocytes. PTPRZ1 labeling demonstrates effective knockdown with shPTPRZ1. β-tubulin is used as a loading control. Download Figure 1-1, TIF file. [file eneuro-13-ENEURO.0386-25.2026-s002.tif]

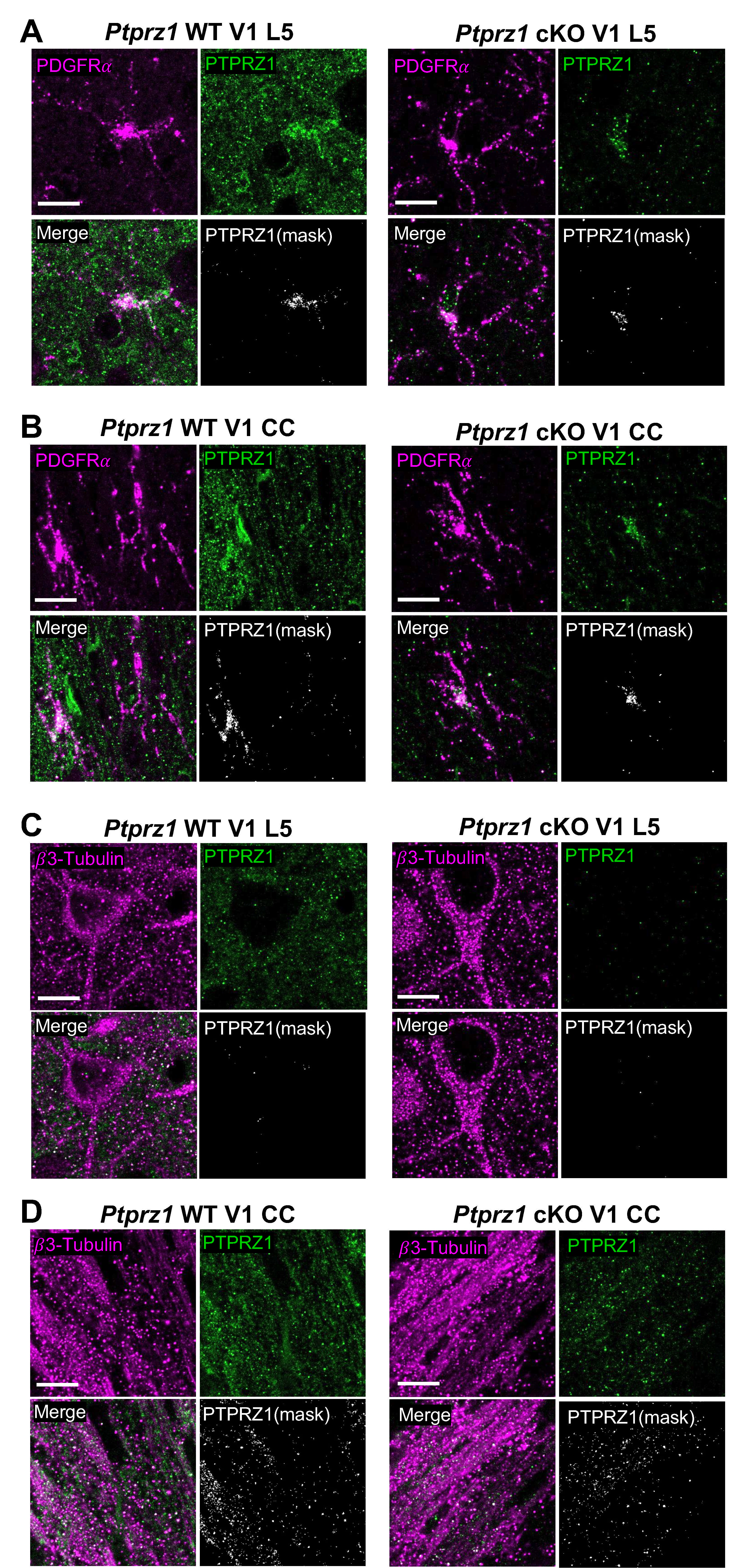

Supplement: Figure 2-1 — Validation of PTPRZ1 expression in OPCs and neurons following conditional Ptprz1 deletion in astrocytes. A-B) High-magnification images of individual V1 L5 (A) and corpus callosum (CC); B) oligodendrocyte precursor cells (OPCs; PDGFRα) from Ptprz1 WT (left) and Ptprz1 cKO (right) mice. Immunolabeling for PDGFRα (magenta) and PTPRZ1 (green) demonstrates high PTPRZ1 expression by OPCs independent of genotype. Binarized masks show PTPRZ1 localization within OPC cell bodies and processes. Scale bars = 10μm. C-D) High-magnification images of neuronal cell bodies and processes (β3-Tubulin) in V1 L5 (C) and CC (D) from Ptprz1 WT (left) and Ptprz1 cKO (right) mice. Immunolabeling for β3-Tubulin (magenta) and PTPRZ1 (green) demonstrates low PTPRZ1 expression by neurons independent of genotype. Binarized masks show PTPRZ1 localization within neuronal cell bodies and proximal processes (C) or axon tracts (D). Scale bars = 10μm. Download Figure 2-1, TIF file. [file eneuro-13-ENEURO.0386-25.2026-s003.tif]

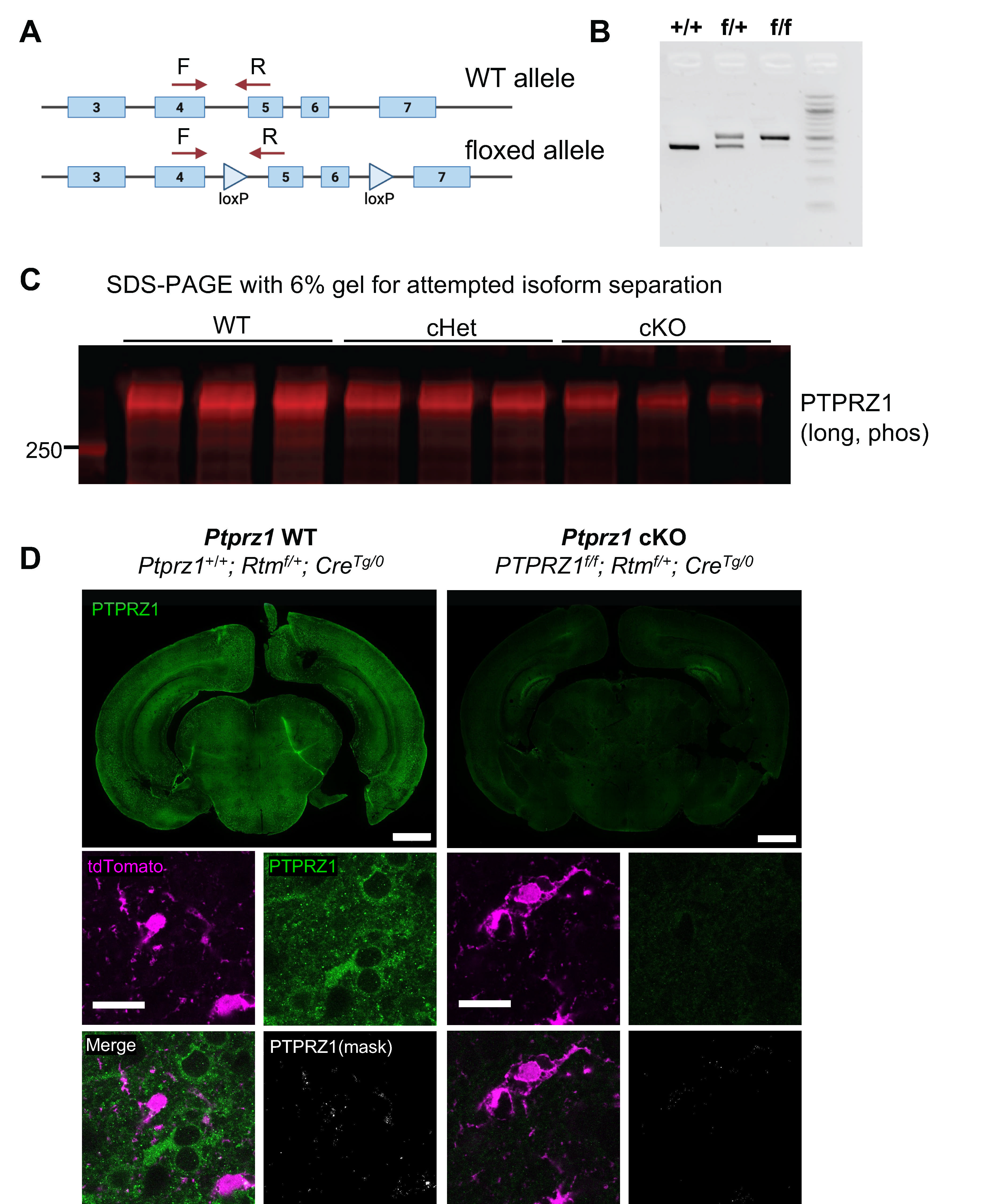

Supplement: Figure 2-2 — Additional validation of Ptprz1 conditional deletion. A) Genotyping strategy for detecting wild-type (WT) and floxed Ptprz1 alleles from mouse genomic DNA. The same forward and reverse primers detect both alleles, with the floxed allele appearing 89 base pairs higher due to the addition of the loxP site. B) Example of PCR products obtained from WT (+/+), f/+, and f/f mice. C) Western blot of PTPRZ1 showed failed separation of long and secreted isoforms on a 6% gel. D) Representative tile scan images of P7 coronal sections containing primary VCX (top) and high-magnification images of individual V1 L5 astrocytes (Td-Tomato; bottom) from Ptprz1 WT and Ptprz1 cKO mice. Immunolabeling for PTPRZ1 (green) and Td-Tomato (magenta) demonstrates effective deletion of PTPRZ1 from astrocytes. Binarized masks show PTPRZ1 localization within astrocyte cell bodies and processes. Overview scale bar 1000 µm, high-magnification scale bar 10 µm. Download Figure 2-2, TIF file. [file eneuro-13-ENEURO.0386-25.2026-s004.tif]

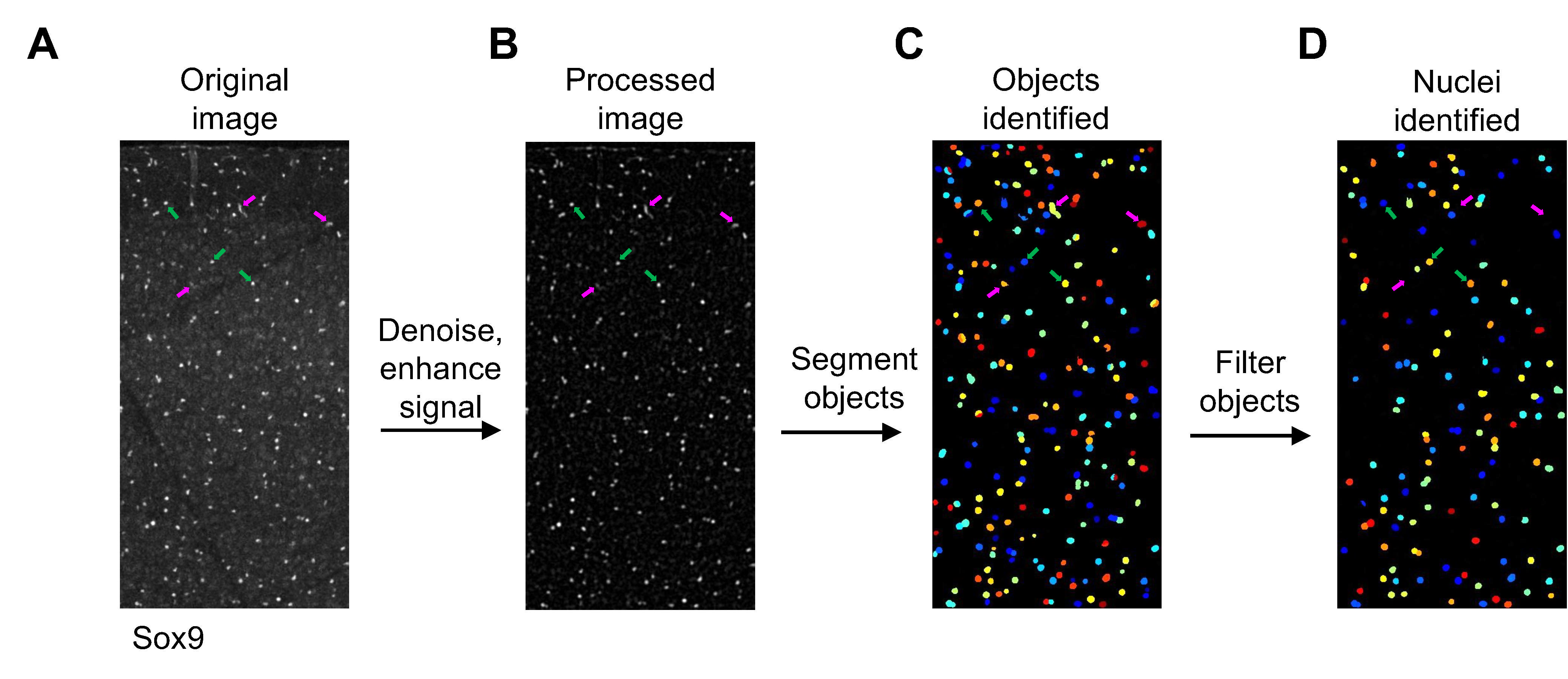

Supplement: Figure 3-1 — Cell counting workflow. A) Representative input image for the cell count pipeline. Cropped grayscale image of visual cortex with Sox9-labeled nuclei. Green arrows denote representative Sox9 + nuclei that will eventually be included in the cell count. Magenta arrows denote representative debris that will eventually be excluded from the cell count. B) Processed input image, after denoising, foreground signal enhancement, and nuclei-specific signal enhancement. Signal separates more clearly from the background, and nuclei appear more distinct from debris, compared to the original image. C) Identified objects segmented from the processed image. Both nuclei and debris are identified as objects. D) Objects representing debris are filtered out. Objects representing Sox9 + nuclei remain and are included in the quantification of Sox9 + cells in the image. Download Figure 3-1, TIF file. [file eneuro-13-ENEURO.0386-25.2026-s005.tif]

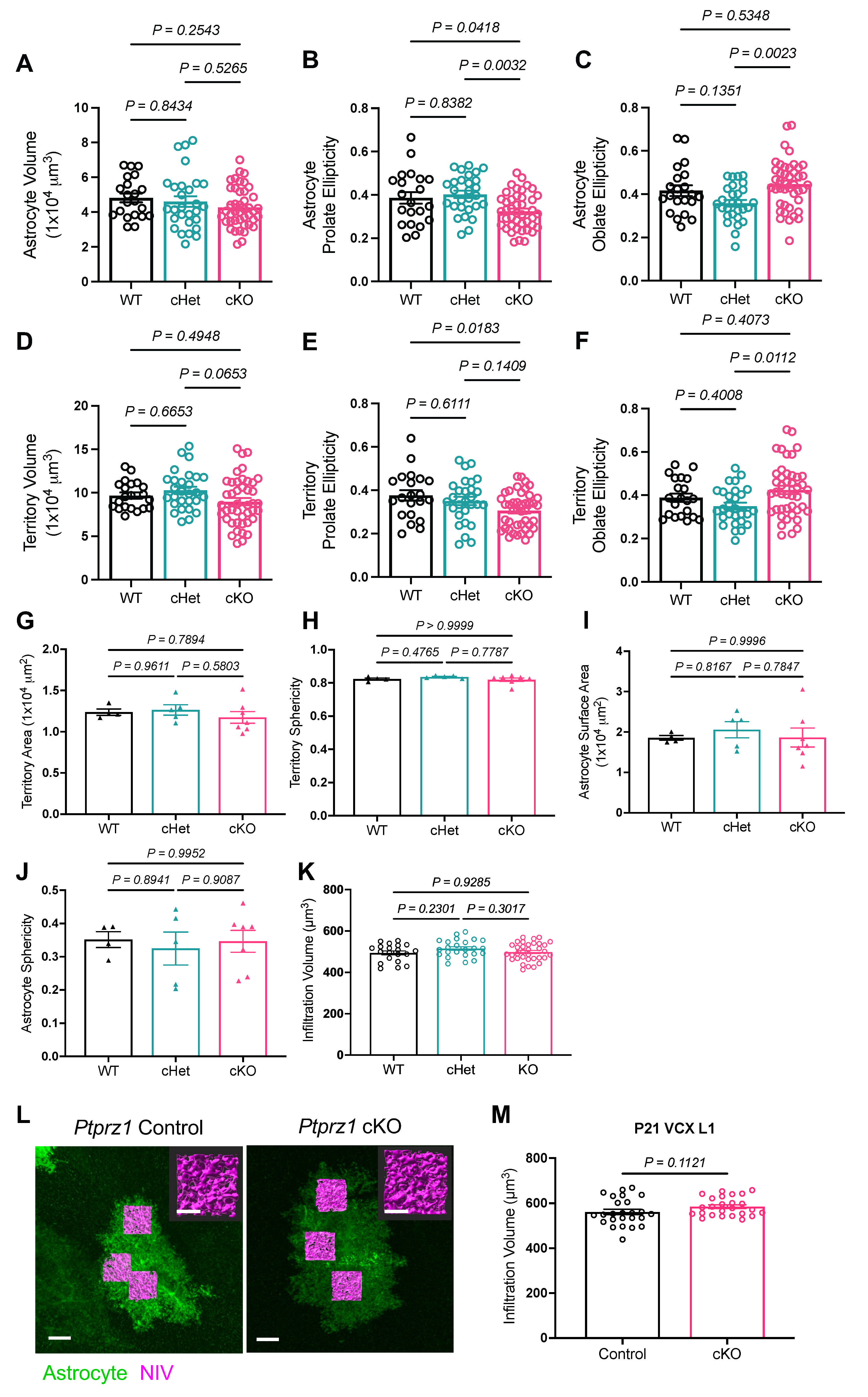

Supplement: Figure 4-1 — Additional 3D morphological analyses of V1 L1 and L5 astrocytes. (A-F) Individual astrocyte statistics represented by open circles for 3D morphology metrics presented in Fig. 4: (A) astrocyte volume, (B) astrocyte prolate ellipticity, (C) astrocyte oblate ellipticity, (D) territory volume, (E) territory prolate ellipticity, and (F) territory oblate ellipticity in P21 V1 L5 astrocytes. For each metric, n = 4 (WT), n = 5 (cHet) and n = 7 (cKO) mice per group, 4-8 cells per mouse. Bars are mean +/- SEM. One-way ANOVA, Tukey’s post-test. (G-J) Additional 3D morphology analyses of: (G) Analysis of territory area (η2 = 0.079), (H) territory sphericity, (I) astrocyte surface area (η2 = 0.0404), and (J) astrocyte sphericity (η2 = 0.0191) in P21 V1 L5 astrocytes. Data presented as subject averages (individual mice represented as triangles; n as in (A-F), 4-8 cells per mouse). Bars are mean +/- SEM. One-way ANOVA, Tukey’s post-test, effect size reported as η2 (G, I-J) or Kruskal-Wallis test, Dunn’s multiple comparisons test (H). K) NIV analysis as in Fig. 4 K for individual astrocytes, represented by open circles. Three ROIs/cell, 4-5 cells/mouse, n as in (A-F). Bars are mean +/- SEM. One-way ANOVA, Tukey’s post-test. L) Representative V1 L1 astrocytes at P21 expressing eGFP-CAAX (green) with Neuropil Infiltration Volume (NIV) reconstructions (magenta; inset scale = 5 µm). Scale bar 10μm. M) NIV analysis for Ptprz1 control (WT + cHet) and cKO astrocytes. Open circles represent individual astrocytes from 11 animals, n = 25 cells per group. Bars are mean +/- SEM. Unpaired t-test. Download Figure 4-1, TIF file. [file eneuro-13-ENEURO.0386-25.2026-s006.tif]

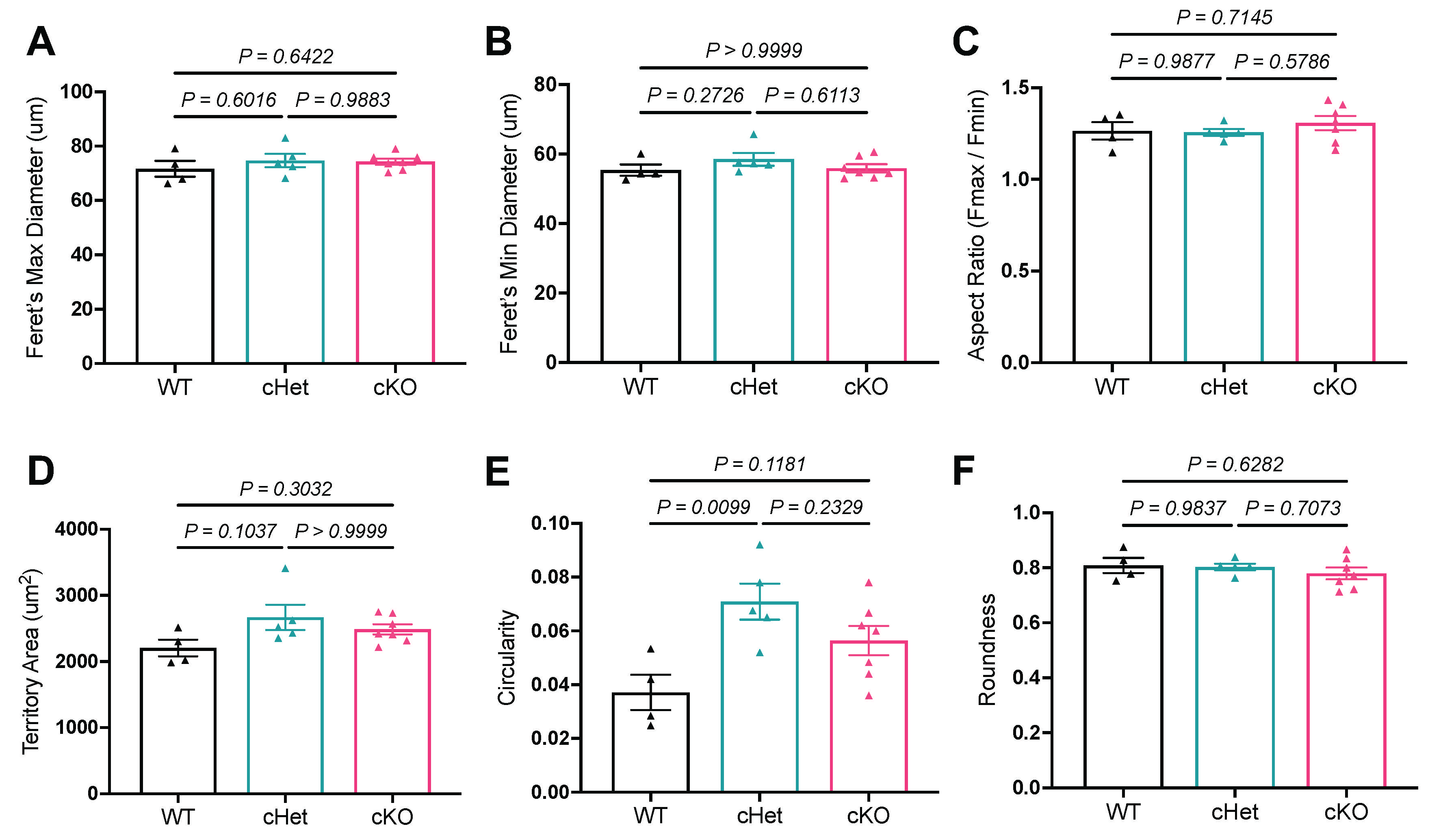

Supplement: Figure 4-2 — Additional 2D morphological analyses of V1 L5 astrocytes. (A-F) 2D morphology analyses of: (A) Feret’s max diameter (η2 = 0.0799), (B) Feret’s min diameter, (C) aspect ratio (Feret Max/Feret Min; η2 = 0.0859), (D) territory area, (E) circularity (η2 = 0.488), and (F) roundness (η2 = 0.0783). Triangles represent subject averages (n = 4 (WT), n = 5 (cHet) and n = 7 (cKO) mice per group, 4-6 cells per mouse). Bars are mean +/- SEM. One-way ANOVA, Tukey’s post-test, effect size reported as η2 (A, C, E-F) or Kruskal-Wallis test, Dunn’s multiple comparisons test (B, D). Download Figure 4-2, TIF file. [file eneuro-13-ENEURO.0386-25.2026-s007.tif]

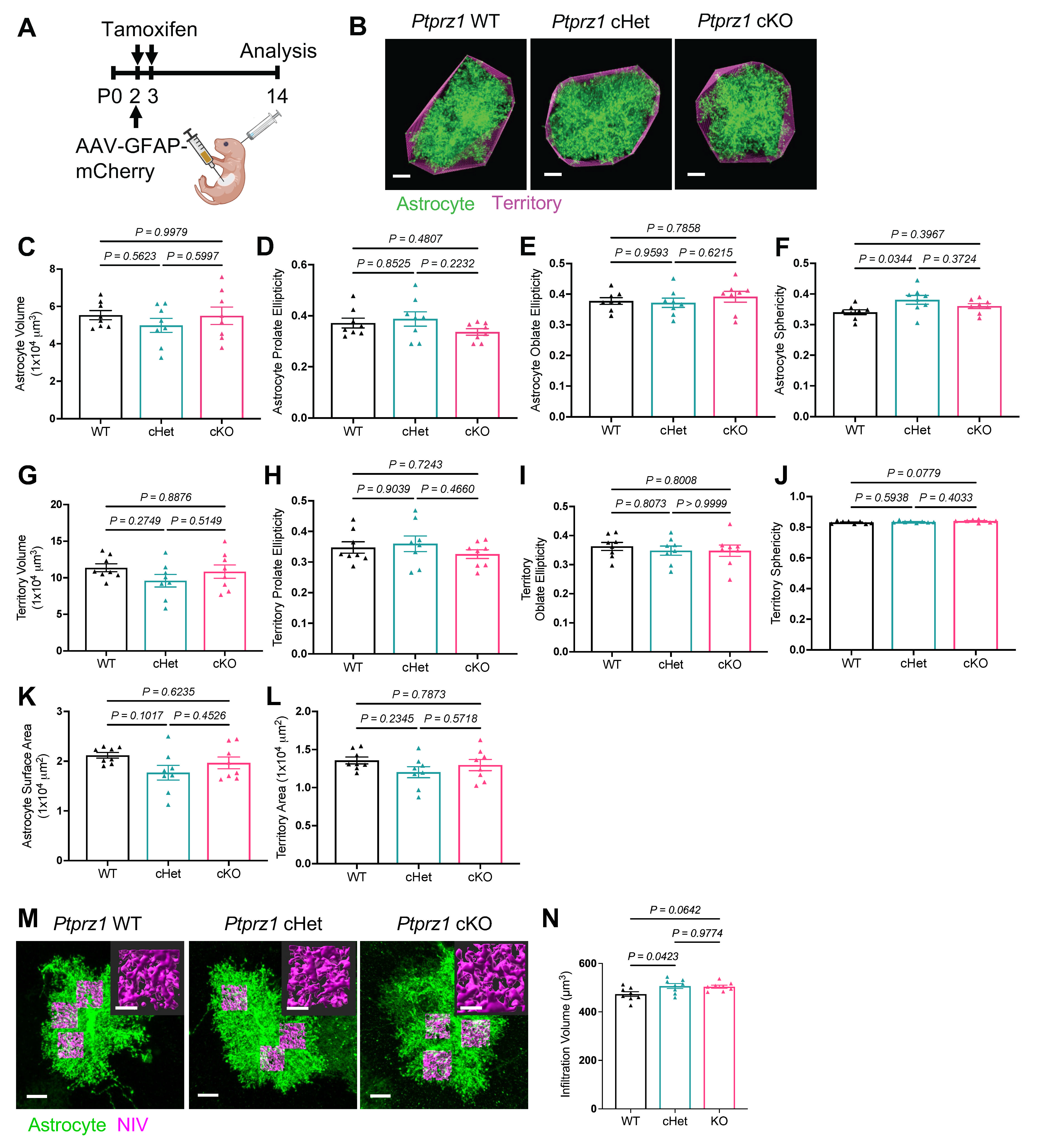

Supplement: Figure 4-3 — Modest morphology differences at P14 in Ptprz1 cHet. A) Tamoxifen administration and AAV injection strategy for Ptprz1 cKO and sparse labeling for detailed morphology analyses. B) V1 L5 astrocytes at P14 expressing mCherry-CAAX in Ptprz1 WT (left), Ptprz1 cHet (center) and Ptprz1 cKO (right) mice. Astrocytes expressing mCherry-CAAX in green; astrocyte territory in magenta. Scale bar, 10μm. (C-L) 3D morphology analyses of: (C) astrocyte sphericity (η2 = 0.258), (D) astrocyte volume (η2 = 0.0606), (E) astrocyte prolate ellipticity (η2 = 0.128), (F) astrocyte oblate ellipticity (η2 = 0.0426), (G) territory sphericity (η2 = 0.202), (H) territory volume (η2 = 0.112), (I) territory prolate ellipticity (η2 = 0.0658), (J) territory oblate ellipticity (η2 = 0.0248), (K) astrocyte surface area (η2 = 0.183), and (L) territory area (η2 = 0.121) in P14 V1 L5 astrocytes. Data presented as subject averages (individual mice represented as triangles; n = 8 mice per group, 5 cells per mouse). Bars are mean +/- SEM. One-way ANOVA, Tukey’s post-test. Effect size reported as η2. M) Representative V1 L5 astrocytes at P14 expressing mCherry-CAAX (green) with Neuropil Infiltration Volume (NIV) reconstructions (magenta; inset scale = 5μm). Scale bar 10μm. (N) NIV analysis for Ptprz1 WT, cHet and cKO astrocytes (η2 = 0.285). Three ROIs/cell, 5 cells/mouse, n as in (C-L). N) Data presented as subject averages as above (C-L). Bars are mean +/- SEM. One-way ANOVA, Tukey’s post-test. Effect size reported as η2. Download Figure 4-3, TIF file. [file eneuro-13-ENEURO.0386-25.2026-s008.tif]

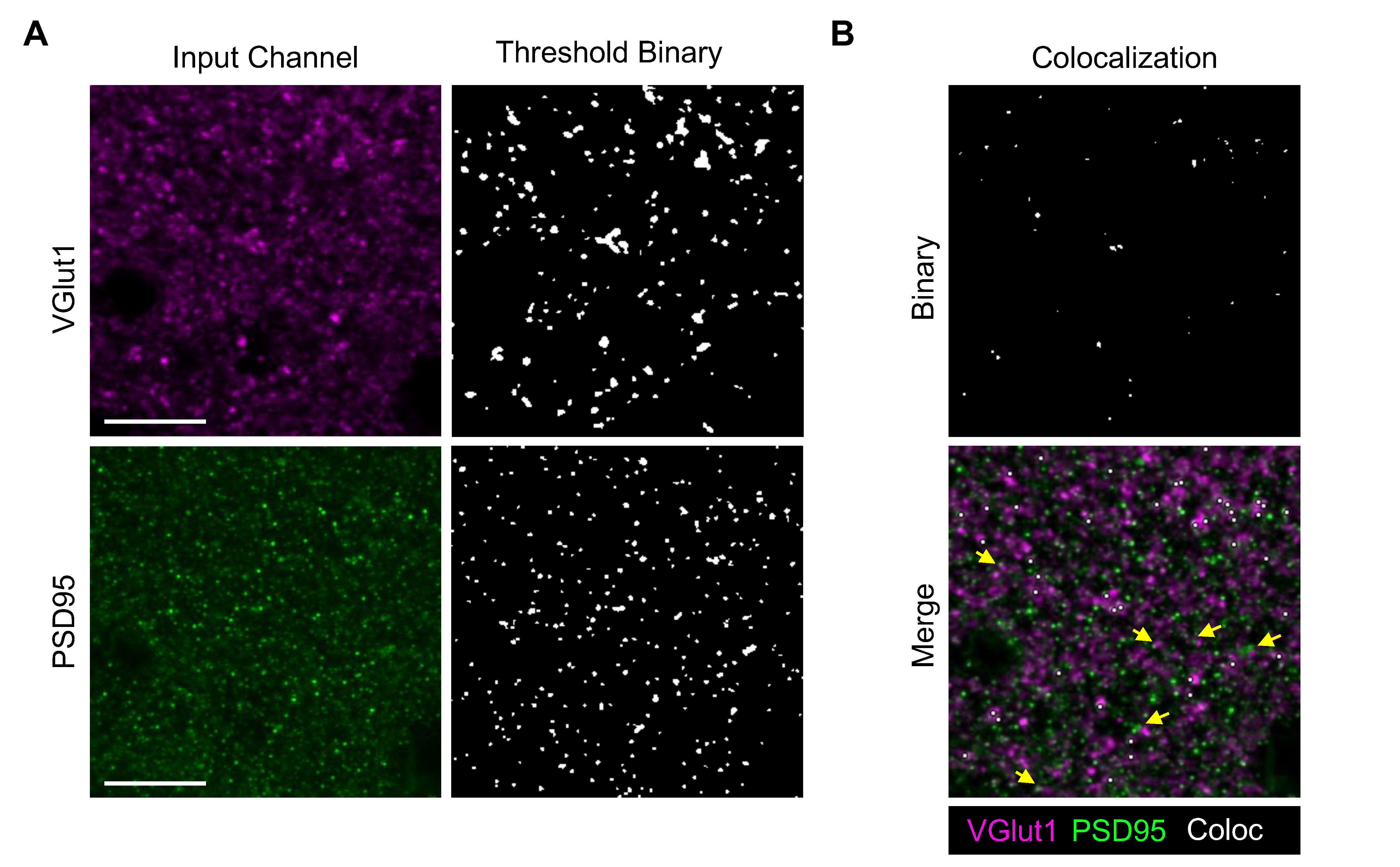

Supplement: Figure 6-1 — Demonstration of thresholding strategy for synaptic marker analysis using Synbot. A) Left: Representative images of VGlut1 (top left, magenta) and PSD95 (bottom left, green) from P21 L1 visual cortex. Scale bar 10 µm. Right: binary images of each channel following thresholding. B) Top: Binary output of co-localized VGlut1 and PSD95 signal. Bottom: Merged output image generated by Synbot that contains merged channels (VGlut1 in magenta, PSD95 in green) and labeling of co-localized puncta (white dots). To demonstrate stringency of this analysis method, yellow arrows have been added to highlight examples of magenta and green puncta that are very close to each other, but do not overlap and are not counted by Synbot. Download Figure 6-1, TIF file. [file eneuro-13-ENEURO.0386-25.2026-s009.tif]

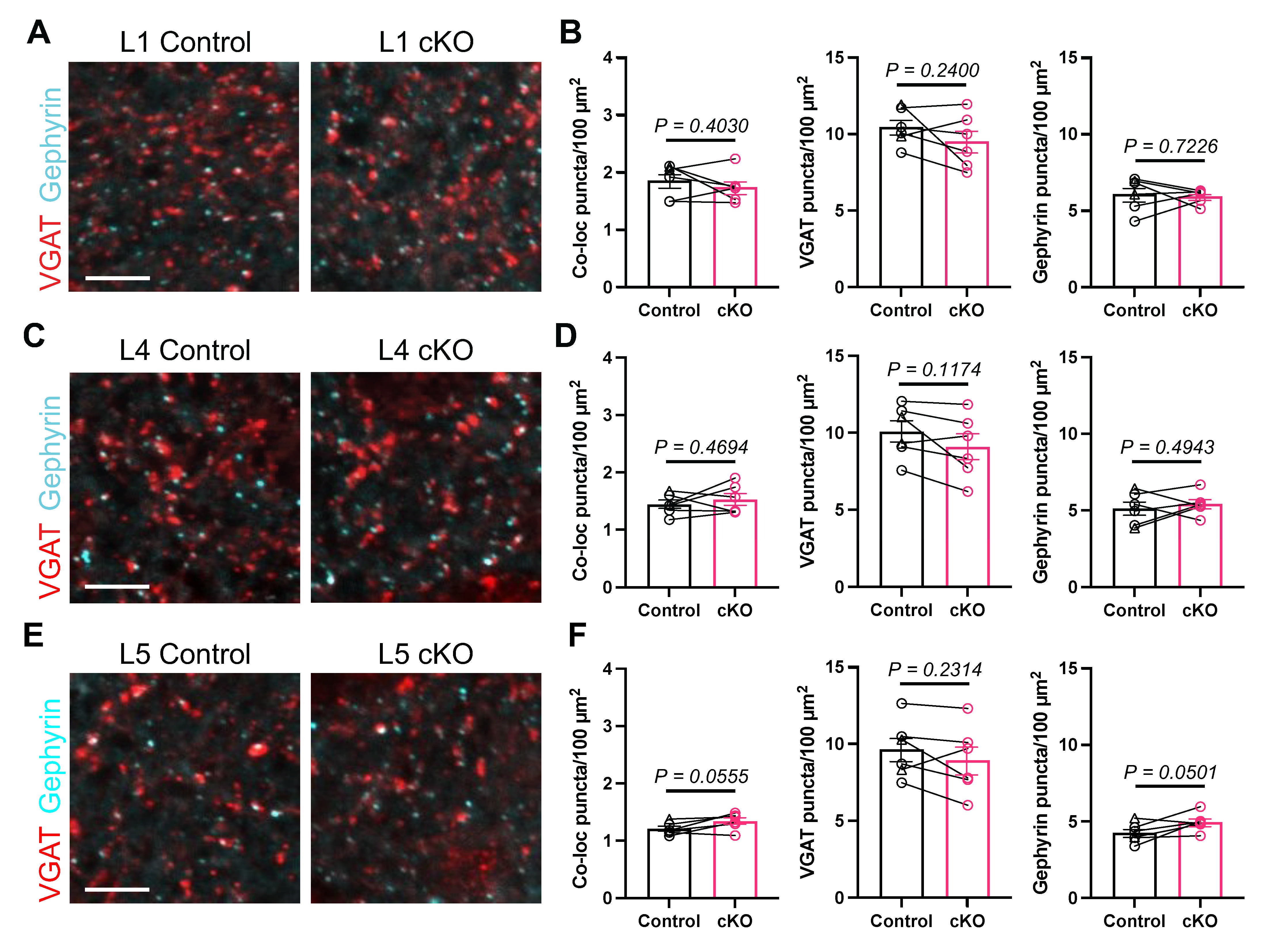

Supplement: Figure 6-2 — Density of co-localized inhibitory synapse markers is unchanged at P21. A) Representative 17.5 µm x 17.5 µm regions of interest (ROIs) labeled with inhibitory synapse markers in V1 L1 at P21. Presynaptic VGAT (red) and postsynaptic marker gephyrin (cyan). Scale bar 5 µm. B) Density of co-localized puncta (left) (d = 0.527, CI [-0.957, 2.011]), VGAT puncta (middle) (d = 0.769, CI [-0.715, 2.253]), and Gephyrin puncta (right) (d = 0.214, CI [-1.270, 1.698]) from L1. C) Representative ROIs labeled with VGAT and gephyrin in L4 and D) quantification of co-localized (d = -0.444, CI [-1.928, 1.040]), VGAT (d = 1.091, CI [-0.393, 2.575]), and gephyrin (d = -0.415, CI [-1.899, 1.069]) puncta density. E) Representative ROIs labeled with VGAT and gephyrin in L5 and F) quantification of co-localized (d = -1.434, CI [-2.918, 0.049]), VGAT (d = 0.786, CI [-0.698, 2.270]), and gephyrin (d = -1.356, CI [-2.839, 0.128]) puncta density. For B, D, and F: n = 6 sex-matched littermate pairs of control and cKO mice. In the control column, a triangle denotes WT mice and circle denotes cHet. Lines connect sex-matched control-cKO littermates. Dots represent per animal averages of 15 images. P-values were calculated using a linear mixed effects model. Effect size reported above as Cohen’s d (d) with 95% Confidence Intervals (CI [lower, upper]). Download Figure 6-2, TIF file. [file eneuro-13-ENEURO.0386-25.2026-s010.tif]
